# The neuroblast timer gene *nubbin* exhibits functional redundancy with gap genes to regulate segment identity in *Tribolium*

**DOI:** 10.1242/dev.199719

**Published:** 2021-08-19

**Authors:** Olivia R. A. Tidswell, Matthew A. Benton, Michael Akam

**Affiliations:** Department of Zoology, University of Cambridge, Cambridge CB2 3EJ, UK

**Keywords:** *Tribolium castaneum*, Gap gene, *nubbin*, *castor*, Hox gene, Neuroblast

## Abstract

The neuroblast timer genes *hunchback*, *Krüppel*, *nubbin* and *castor* are expressed in temporal sequence in neural stem cells, and in corresponding spatial sequence along the *Drosophila* blastoderm. As canonical gap genes, *hunchback* and *Krüppel* play a crucial role in insect segmentation, but the roles of *nubbin* and *castor* in this process remain ambiguous. We have investigated the expression and functions of *nubbin* and *castor* during segmentation in the beetle *Tribolium*. We show that *Tc-hunchback*, *Tc-Krüppel*, *Tc-nubbin* and *Tc-castor* are expressed sequentially in the segment addition zone, and that *Tc-nubbin* regulates segment identity redundantly with two previously described gap/gap-like genes, *Tc-giant* and *Tc-knirps*. Simultaneous knockdown of *Tc-nubbin*, *Tc-giant* and *Tc-knirps* results in the formation of ectopic legs on abdominal segments. This homeotic transformation is caused by loss of abdominal Hox gene expression, likely due to expanded *Tc-Krüppel* expression. Our findings support the theory that the neuroblast timer series was co-opted for use in insect segment patterning, and contribute to our growing understanding of the evolution and function of the gap gene network outside of *Drosophila*.

## Abstract (precis)

**Summary:***nubbin* and the gap genes *knirps* and *giant* redundantly repress *Krüppel* expression during segmentation. Simultaneous knockdown of all three genes causes ectopic *Krüppel* expression and loss of abdominal segment identity.

## INTRODUCTION

The gap gene network of *Drosophila* is arguably one of the best characterised gene regulatory networks in developmental biology. Gap genes mediate two central processes in *Drosophila* segmentation – the formation of segment boundaries and the assignment of segment identities – through direct regulation of pair-rule and Hox genes, respectively (reviewed by Jaeger, 2011). Homologs of many *Drosophila* gap genes also regulate segment patterning in other insect species (Bucher and Klingler, 2004; Cerny et al., 2005; Liu and Kaufman, 2004b; Liu and Patel, 2010; Marques-Souza et al., 2008; Mito et al., 2005, 2006). Recent attention has therefore turned to understanding how gap genes interact and function outside of *Drosophila*, in order to better understand the origins and evolution of this important gene network.

**Fig. 1.**
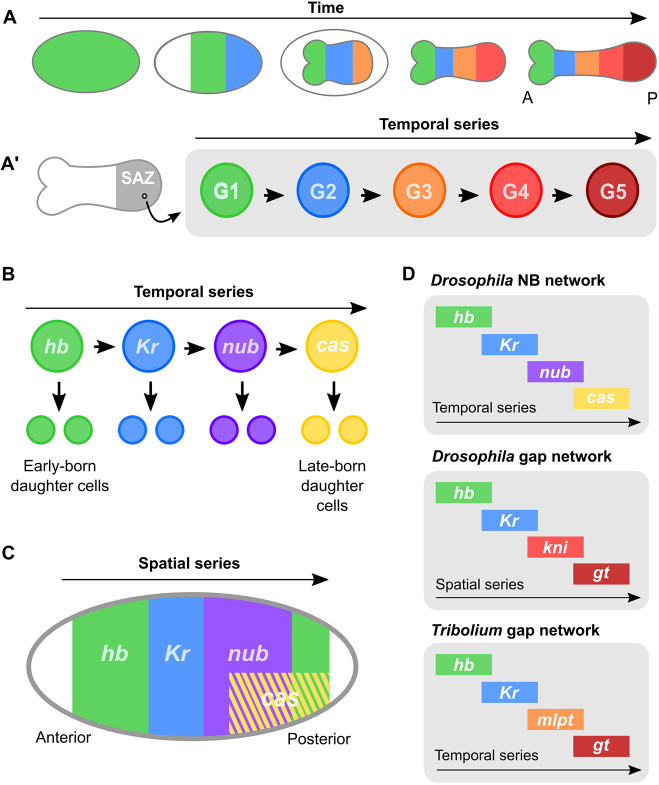
**Parallels between the gap gene network and neuroblast timer network in insects.** (A) Gap gene expression during *Tribolium* development. Gap gene expression domains emerge sequentially from the posterior of the embryo (the SAZ) in this sequentially segmenting insect (A). A, anterior; P, posterior. Cell lineages persisting in the SAZ express a temporal sequence of gap genes (A′). G1-5, gap genes 1-5. (B) The neuroblast timer sequence in *Drosophila*. The genes *hb*, *Kr*, *nub* and *cas* are expressed sequentially in embryonic neuroblasts, where they regulate assignment of daughter cell fates. (C) Expression of the neuroblast timer genes along the anterior-to-posterior (AP) axis of the *Drosophila* blastoderm. The spatial sequence is similar to the temporal sequence in neuroblasts. (D) The *Drosophila* neuroblast (NB) timer network and the canonical *Drosophila* and *Tribolium* gap gene networks comprise overlapping, but not identical, sets of genes.

In *Drosophila*, the gap genes are thought of as markers for spatial domains, regulated initially by gradients of maternal factors, and then by cross-regulation within the gap gene network itself (Jaeger, 2011). However, recent work, particularly in the red flour beetle *Tribolium castaneum*, leads to a rather different way of viewing these same genes. In *Tribolium* and other sequentially segmenting insects, segments are added progressively, from anterior to posterior, from a segment addition zone (SAZ) at the posterior of the extending germ band (Clark et al., 2019). Gap genes are sequentially activated in the SAZ, so that cells persisting in this region experience a temporal sequence of gap gene expression (Boos et al., 2018; Zhu et al., 2017) (Fig. 1A). As each cell exits the SAZ, its gap gene expression is stabilised (Zhu et al., 2017), creating a spatial pattern of gap gene expression along the anterior-posterior axis of the trunk. The gap genes may therefore provide a timer for the maturation of cells with different axial identities from the segment addition zone (Bucher and Klingler, 2004; Cerny et al., 2005; Clark et al., 2019).

This model of the gap gene network has many similarities to the neuroblast timer network that regulates embryonic neural patterning in insects (Clark et al., 2019; Doe, 2017). The insect nervous system is produced by neural stem cells known as neuroblasts, each of which gives rise to a range of different cell types in a stereotyped order. In embryonic neuroblasts of *Drosophila*, this order is directed by the sequential expression of the neuroblast timer genes *hunchback* (*hb*), *Krüppel* (*Kr*), *nubbin* (*nub*), *castor* (*cas*) and *grainyhead* (*grh*) (reviewed by Brody and Odenwald, 2005) (Fig. 1B). Homologues of *hb*, *nub* and *cas* are expressed in the same relative order in some vertebrate neural stem cells, where they regulate the fate of neurons derived from their progeny (Alsiö et al., 2013; Elliott et al., 2008; Javed et al., 2020; Mattar and Cayouette, 2015; Mattar et al., 2018). This suggests that the roles of these genes in neural development are deeply conserved.

Parallels between the neuroblast timer series and the gap gene network have long been noted (Isshiki et al., 2001; Peel et al., 2005), giving rise to the hypothesis that elements of the neuroblast timer network may have been co-opted from neuroblasts for use in insect axial patterning (Peel et al., 2005). The first two genes in the neuroblast timer series, *hb* and *Kr*, are also canonical gap genes in *Drosophila* and *Tribolium* (Boos et al., 2018; Cerny et al., 2005; Marques-Souza et al., 2008). However, the next two genes in the neuroblast timer series, *nub* and *cas*, are not canonical gap genes in *Drosophila* (Jaeger, 2011). The canonical gap genes acting posteriorly to *Kr* in *Drosophila* – *Dm-knirps* (*kni*) and *Dm-giant* (*gt*) *–* and in *Tribolium – Tc-gt* and *Tc-mille-pattes –* are not components of the neuroblast timer series (Fig. 1D).

Although *nub* and *cas* are not canonical gap genes, they do show some intriguing similarities to gap genes. *Dm-nub* (and its closely linked paralogue *Dm-pdm2*) and *Dm-cas* are expressed in the *Drosophila* blastoderm during segment patterning, in spatial domains that follow in sequence behind *Dm-hb* and *Dm-Kr* (Cockerill et al., 1993; Isshiki et al., 2001) (Fig. 1C). Ectopic expression of *Dm-nub* or *Dm-pdm2* results in gap-like segment deletions (Cockerill et al., 1993); however, neither gene appears to regulate the canonical gap genes (Cockerill et al., 1993), and deletion of both genes generates only incompletely penetrant and variable segment fusions (Cockerill et al., 1993; Ma et al., 1998). *Dm-cas* is not known to have any role in segmentation (e.g. Mellerick et al., 1992).

Data from sequentially segmenting insects has identified further parallels. A homologue of *nub* is necessary for the correct specification of abdominal segment identity in the bug *Oncopeltus* (Hrycaj et al., 2008), although not in the cricket *Acheta* (Turchyn et al., 2011). In *Tribolium*, *Tc-nub* and *Tc-cas* are also expressed in the segment addition zone (Biffar and Stollewerk, 2014), but parental RNA interference has failed to identify any role for *Tc-nub* in segmentation (E. Raymond and A. Peel, personal communication); functional analyses have not been carried out for *Tc-cas*.

In this article, we examine whether *Tc-nub* and *Tc-cas* form part of a temporal sequence of gene expression during segmentation in *Tribolium*, and ask whether either regulates segment addition or the assignment of segment identities. Our functional analyses demonstrate a clear role for *Tc-nub* in the assignment of abdominal segment identity. This role is partially redundant with that of other abdominal gap genes, explaining why it has not been identified previously. Our findings strengthen the hypothesis that elements of the gap gene network may have been recruited for a timing role in axial patterning from a pre-existing role in neural development.

## RESULTS

### The neuroblast timer genes are expressed sequentially in the SAZ

**Fig. 2.**
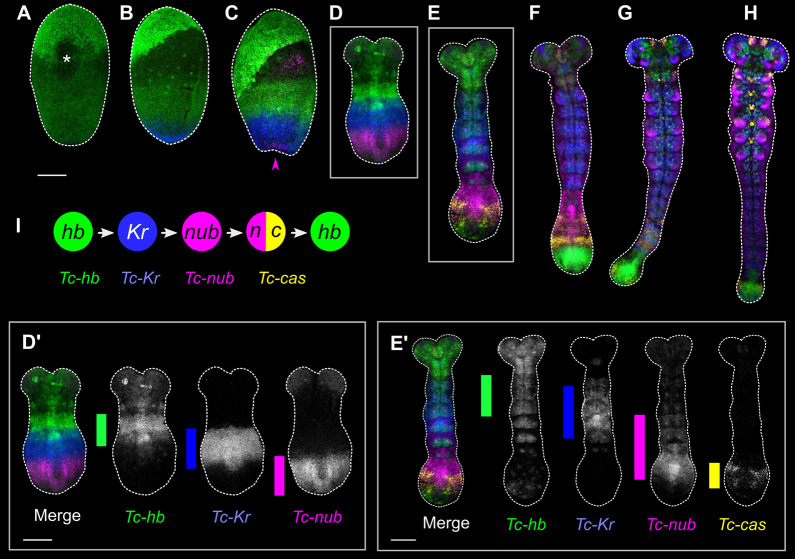
**Expression of the neuroblast timer genes during segment addition in *Tribolium*.** (A-H) Expression of *Tc-hb*, *Tc-Kr*, *Tc-nub* and *Tc-cas* in embryos spanning the course of segment addition, from the differentiated blastoderm stage (A) to the end of segment addition (H). The asterisk in A highlights damage to the embryo. The magenta arrowhead in C indicates the emergence of *Tc-nub* expression in the posterior pit. D′ and E′ show greyscale images of the channels in embryos D and E. Coloured bars highlight the extent of the ectodermal ‘gap-like’ expression domain of each gene along the AP axis. In D′, the ‘gap’ phase of *Tc-hb* expression has almost entirely faded, and the staining visible is mostly mesodermal expression. The anterior of each embryo is towards the top of the figure; ventral is along the vertical midline of each image, except in B and C, where it is angled towards the right. (I) A summary of the gene expression states experienced by cells in the SAZ, inferred from expression dynamics in A-H. Scale bars: 100 μm.

We first examined whether the genes of the neuroblast timer series are expressed in temporal order in the SAZ of *Tribolium* during segment addition. We used hybridisation chain reaction (HCR) RNA *in situ* hybridisation (ISH) (Choi et al., 2018) to examine the expression patterns of *Tc-hb*, *Tc-Kr*, *Tc-nub* and *Tc-cas* in *Tribolium* embryos spanning the stages of segment addition [8-22 h after egg lay (AEL) at 30°C]. We found that these four genes are expressed sequentially in the SAZ in largely the same order as they are expressed in neuroblasts, and that this sequential expression results in their being expressed in spatial order along the anterior-to-posterior (AP) axis of the embryonic trunk (Fig. 2). *Tc-hb* mRNA is initially distributed broadly across the blastoderm (Wolff et al., 1995) (Fig. 2A), becoming lost from the posterior tip of the embryo as *Tc-Kr* expression emerges (Boos et al., 2018) (Fig. 2B). *Tc-nub* becomes expressed at the posterior tip of the embryo shortly afterwards, correlating with loss of *Tc-Kr* expression in the same region (Fig. 2C,D). *Tc-cas* becomes expressed in the SAZ midway through germband extension, in a domain overlapping the posterior of the *Tc-nub* domain (Fig. 2E,F). Finally, a second domain of *Tc-hb* becomes expressed in the posterior SAZ and remains expressed in the SAZ until the end of segment addition (Fig. 2E-H). This re-expression of *hb* after *cas* is not observed during neurogenesis in either *Drosophila* or *Tribolium* (Biffar and Stollewerk, 2014; Doe, 2017), so is a distinctive feature of the SAZ. Each of these genes is also expressed in the neurectoderm and/or neuroblasts in differentiating segments, as well as in the tissue at the extreme posterior of the embryo – the presumptive hindgut epithelium (Benton, 2018) – after segmentation is complete (Fig. S1).

### Expression of *Tc-nub* and *Tc-cas* in relation to segment patterning

To characterise the expression dynamics of *Tc-nub* and *Tc-cas* in more detail, we next examined the expression of both genes against expression of the segment polarity gene *Tc-wingless* (*Tc-wg*) (Nagy and Carroll, 1994) in embryos spanning the course of segment addition. *Tc-wg* stripes form sequentially in the trunk over the course of segment addition and can therefore be used as a proxy for developmental stage. Each *Tc-wg* stripe marks the posterior boundary of a parasegment (PS), and has been assigned a number that reflects its relationship to that parasegment (e.g. wg6 sits at the posterior of PS6; the first trunk *Tc-wg* stripe is designated wg0, as it sits at the posterior of PS0).

**Fig. 3.**
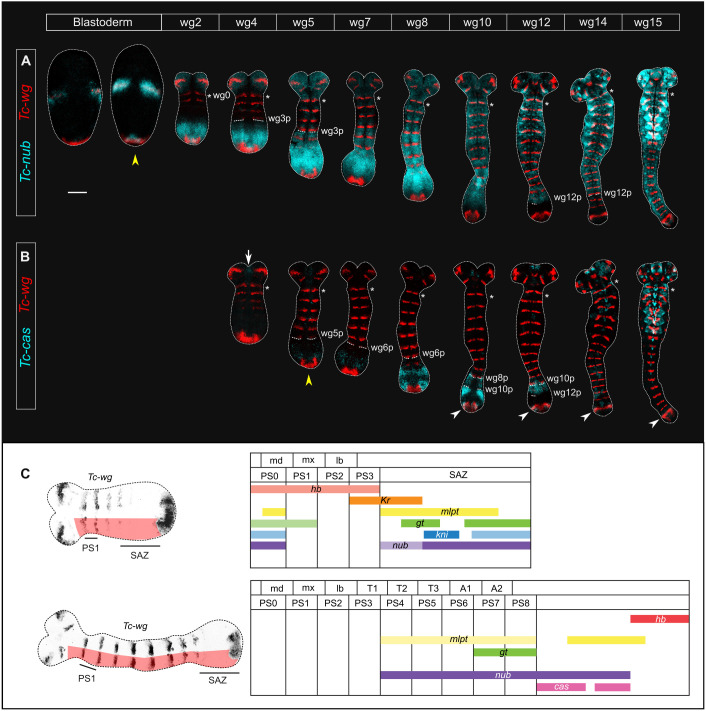
**Expression of *Tc-nub* and *Tc-cas* in *Tribolium* embryos during segment addition, using *Tc-wg* as a segmental marker.** (A,B) Expression of *Tc-nub* (A) and *Tc-cas* (B) over the course of segment addition. Column headers indicate the identity of the most recently formed *Tc-wg* stripe as a proxy for developmental stage. The first column, labelled ‘Blastoderm’, comprises blastoderm-stage embryos that are yet to form any trunk *Tc-wg* stripes. Images in the same column come from the same embryo. Asterisks mark the first *Tc-wg* stripe to form in the trunk (wg0). wg3-12p*,* the posterior boundaries of wg3-12. Yellow arrowheads mark the onset of *Tc-nub* and *Tc-cas* expression in the segment addition zone. The white arrow and arrowheads indicate *Tc-cas* expression in the developing labrum and overlapping the terminal domain of *Tc-wg* expression, respectively. The anterior of each embryo is towards the top of the figure, and ventral is along the vertical midline of each image. (C) Diagrams of *Tc-nub* and *Tc-cas* expression relative to the expression of other *Tribolium* gap genes, based on published descriptions (Bucher and Klingler, 2004; Cerny et al., 2008; Marques-Souza et al., 2008; Peel et al., 2013; Savard et al., 2006; Wolff et al., 1995), at two stages of segment addition. Diagrams span from a short distance anterior to wg0 (i.e. within PS0) to the anterior boundary of the terminal domain of *Tc-wg* (as indicated with red shading on reference embryos to the left). md, mandibular segment; mx, maxillary segment; lb, labial segment; T1-T3, thoracic segments 1-3; A1-A2, abdominal segments 1-2; PS0-8, parasegments 0-8. The anterior of each embryo is to the left and ventral is along the horizontal midline of each image. Scale bar: 100 μm.

*Tc-nub* is expressed at the late blastoderm stage in two patches overlying the ocular *Tc-wg* stripes, and is first expressed at the posterior pole shortly afterwards (Fig. 3A). By the time wg2 has formed, the embryo has condensed to form a germband, and the posterior domain of *Tc-nub* expression has expanded to encompass the posterior one-third of the SAZ (Fig. 3A). The anterior border of this broad gap-like domain abuts wg3 in the ectoderm, but is shifted posteriorly in the mesoderm, abutting wg5 (Fig. S2). Ectodermal expression is weaker anteriorly, and stronger in the posterior SAZ. After the formation of wg6, *Tc-nub* expression begins to fade in the posterior SAZ, and the posterior boundary of *Tc-nub* eventually overlaps with the posterior boundary of wg12 (wg12p; Fig. 3A). *Tc-nub* is therefore expressed in the SAZ during the patterning of PS4-PS12 (posterior compartment of T1 to anterior compartment of A7, inclusive). This overlaps extensively with the expression domains of the gap genes *Tc-mlpt* and *Tc-gt*, and the gap-like gene *Tc-kni* (Fig. 3C).

*Tc-cas* expression is not detectable in the embryo until after the germband has formed. As wg4 and wg5 are forming, *Tc-cas* becomes expressed weakly first in the primordium of the labrum and then in the SAZ (Fig. 3B). Expression in the SAZ is excluded from the mesoderm (Fig. S2). The anterior border of this domain abuts the posterior boundary of wg6 (Fig. 3B). By the time wg8 has formed, expression of *Tc-cas* in the SAZ becomes modulated in a pair-rule pattern; the strongest domains of expression appear to overlap the primordia for PS9 and PS11 immediately after the formation of wg10 (Fig. 3B). Expression of *Tc-cas* subsequently fades in the posterior SAZ and its posterior boundary eventually overlaps with the posterior boundary of wg12 (wg12p; Fig. 3B). This means that *Tc-cas* is expressed in the SAZ during the patterning of PS7-12 (posterior compartment of A3 to anterior compartment of A7, inclusive), overlapping *Tc-nub* and *Tc-mlpt* (Fig. 3C). In maturing segments outside of the SAZ, *Tc-cas* expression fades and is lost (Fig. 3B). There is an additional domain of *Tc-cas* that forms after the emergence of wg10, overlapping the posterior terminal domain of *Tc-wg* (Fig. 3B).

*Tc-nub* later becomes broadly expressed in the ectoderm outside of the SAZ, with slightly stronger expression in the developing neurectoderm (Biffar and Stollewerk, 2014). Both *Tc-nub* and *Tc-cas* are expressed in neuroblasts (Biffar and Stollewerk, 2014) and the limb buds (Fig. 3A,B).

### *Tc-nub*, but not *Tc-cas*, influences segment identity

We next aimed to determine whether *Tc-nub* or *Tc-cas* have a role in axial patterning in *Tribolium*. To do this, we knocked down the expression of each gene by parental and embryonic RNA interference (pRNAi and eRNAi, respectively).

**Fig. 4.**
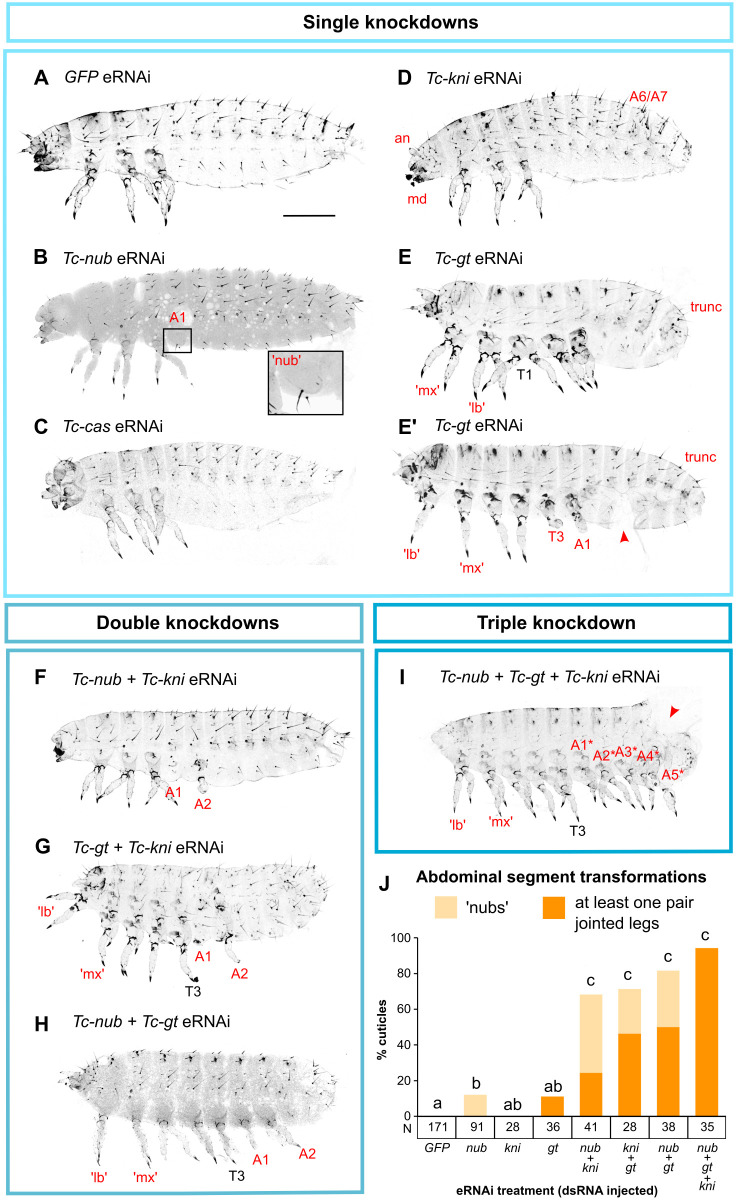
***Tc-nub* acts redundantly with *Tc-gt* and *Tc-kni* to regulate abdominal segment identity.** (A) Control embryos injected with *GFP* dsRNA (2 μg/μl) displayed wild-type abdominal segment morphology. (B) Embryos injected with *Tc-nub* dsRNA (2 μg/μl) sometimes formed cuticular protrusions (‘nubs’, magnified in the inset) on the first abdominal segment (A1). (C) Embryos injected with *Tc-cas* dsRNA (2 μg/μl) showed no consistent defects in cuticular morphology. This specific embryo displays head defects that were common in all treatments, probably resulting from the injections at the anterior pole of the embryo. (D) Embryos injected with *Tc-kni* dsRNA (2 μg/μl) frequently lacked antennal (an) and/or mandibular (md) segments, and displayed disrupted segment patterning in the posterior abdomen (A5-A8). (E,E′) Embryos injected with *Tc-gt* dsRNA (2 μg/μl) frequently formed thoracic legs in the place of maxilla (mx) and labium (lb), and displayed posterior truncation of the abdomen (trunc). A small percentage of injected embryos also developed ectopic legs on segment A1 (E′). (F-H) Embryos injected with any two of *Tc-nub*, *Tc-gt* and *Tc-kni* dsRNAs (1 μg/μl each) frequently formed cuticular protrusions (nubs) and/or ectopic legs (with joints and/or claws) on segments A1 and/or A2. (I) Embryos injected with *Tc-nub*, *Tc-gt* and *Tc-kni* dsRNA (1 μg/μl each) formed ectopic legs on the majority of abdominal segments. The red arrowhead indicates damage to the cuticle sustained during dissection from the eggshell. The asterisks in I indicate that these segment assignments are estimates, based on our understanding of head segment fate in triple knockdowns (Fig. S8). The cuticles in F-I also display head defects consistent with the repression of *Tc-gt* and/or *Tc-kni* expression. For all cuticles, anterior is to the left and dorsal is to the top. (J) A bar graph summarising the frequency of ‘weak’ and ‘strong’ abdominal segment transformations (displaying nubs or jointed/clawed legs, respectively) following eRNAi treatments. A Bayesian logistic regression of abdominal transformation frequency on eRNAi treatment indicated that eRNAi treatments differed significantly in their odds of generating abdominal transformations [χ^2^ (d.f.=7)=314.7, *P*<2.2×10^−16^]. A Tukey post-hoc test was used to determine significant differences between groups, indicated as the letters on top of each column; treatments marked with different letters are significantly different from each other at the *P*<0.02 level. The number of cuticles examined from each treatment is indicated in the row labelled ‘N’, below the *x*-axis. Additional data are provided in Table S2. Scale bar: 200 μm.

We found that pRNAi and eRNAi against *Tc-nub* (2 μg/μl dsRNA) resulted in a subtle abdominal segment transformation in a small percentage of the embryos that survived to the point of cuticle formation. Specifically, 2.9% (pRNAi) and 12.1% (eRNAi) of cuticles displayed a ‘nub’ (an ectopic, ventrolateral protrusion of cuticle, lacking joints or claws) on either side of the first abdominal segment, A1 (Fig. 4B, Tables S1 and S2). This phenotype was never observed in *GFP* pRNAi or eRNAi controls (Tables S1 and S2; Fisher's exact tests: *P*=0.02798 and *P*=5.853×10^−6^, respectively). Similar nubs form following pRNAi against *Tc-abdominal-A* (*Tc-abd-A*, also known as *Tc-Abdominal*; Stuart et al., 1993), and have been interpreted as homeotic transformations of the posterior compartment of an abdominal segment to the posterior compartment of the third thoracic segment, T3 (Lewis et al., 2000). This would make each nub developmentally akin to the posterior compartment of a thoracic leg. We examined the expression of *Tc-abd-A* after *Tc-nub* pRNAi, and found that most of the embryos examined (7/9) showed a downregulation of *Tc-abd-A* expression in the anterior of PS7, which gives rise to A1p (Fig. S3).

Neither pRNAi nor eRNAi against *Tc-cas* had any consistent effects on cuticular morphology or on segment patterning in embryos (*n*=116 and 89, respectively; Fig. 4C and Tables S1 and S2).

Intriguingly, we found that pRNAi, but not eRNAi, against either *Tc-nub* or *Tc-cas* significantly reduced the proportion of eggs that developed to the stage of cuticle formation compared with GFP injection controls (Fig. S4, Tables S1 and S2). Only ∼40-45% of eggs developed cuticle after 1 μg/μl *Tc-nub* or *Tc-cas* pRNAi, compared with 83% of eggs in *GFP* controls (Fisher's exact test: *P*<2.2×10^−6^). *Tc-nub* and *Tc-cas* are both expressed in ovarioles of adult female *Tribolium* (Fig. S5) and may therefore have roles in oogenesis or early embryogenesis. *Dm-cas* is known to be required for the proper formation of follicular cells in *Drosophila* (Chang et al., 2013) but *Dm-nub* does not seem to be expressed in ovaries (Celniker et al., 2009).

We also found that those embryos that do develop to the stage of cuticle formation after pRNAi against *Tc-nub* or *Tc-cas* are significantly less likely to hatch than *GFP* controls, despite their relatively normal external morphology (Fig. S4, Table S1). Specifically, 8-12% of embryos that develop cuticle after 1 μg/μl *Tc-nub* or *Tc-cas* pRNAi go on to hatch, compared with 94% in *GFP* controls (Fisher's exact test: *P*<2.2×10^−6^). The failure of otherwise ‘normal’ larvae to hatch could be a result of defects in the nervous system. Both *Tc-nub* and *Tc-cas* are expressed in neuroblasts in *Tribolium* (Biffar and Stollewerk, 2014), and *Dm-cas* mutants with otherwise normal cuticles also fail to hatch, presumably because of disruption to the nervous system (Mellerick et al., 1992). Together, our data show that *Tc-nub* and *Tc-cas* are likely involved in oogenesis and neurogenesis, and that *Tc-nub* affects specification of segment identity.

### *Tc-nub* acts redundantly with *Tc-gt* and *Tc-kni* to regulate abdominal segment identity

The spatially restricted and weakly penetrant homeotic phenotype observed after *Tc-nub* RNAi contrasts with the expression of this gene across the majority of the abdomen. We hypothesised that the function of *Tc-nub* might be obscured in RNAi experiments by redundancy with co-expressed genes. Two promising candidate genes for redundant function are *Tc-giant* (*Tc-gt*) and *Tc-knirps* (*Tc-kni*), both of which are transiently co-expressed with *Tc-nub* in the SAZ (Fig. 3C, Fig. S6). *Tc-gt* is considered a gap gene in *Tribolium*, as its knockdown affects thoracic segment identity and abdominal segment formation (Bucher and Klingler, 2004). In contrast, *Tc-kni* is not considered to be a gap gene, as its knockdown results in the deletion of only one segment boundary in the head, with no effects on segment identity (Cerny et al., 2008; Peel et al., 2013).

To determine whether *Tc-nub* acts redundantly with *Tc-gt* and/or *Tc-kni* to regulate abdominal segment patterning, we performed single-, double- and triple-knockdowns of these genes. We used eRNAi to avoid any possible negative effects of parental *Tc-nub* knockdown on oogenesis. Single knockdowns of *Tc-kni* and *Tc-gt* produced phenotypes largely consistent with previous reports (Bucher and Klingler, 2004; Cerny et al., 2008; Peel et al., 2013) (Fig. 4D-E′). The notable exception was that 11% of cuticles formed after *Tc-gt* eRNAi also displayed disrupted leg formation on segment T3 and ectopic legs, similarly disrupted, on segment A1 (Fig. 4E′, Table S2). This difference may be due to eRNAi causing stronger knockdown phenotypes than pRNAi, as has been observed previously (Benton et al., 2019).

While knockdown of *Tc-nub* or *Tc-gt* alone resulted in only a low frequency of homeotic transformations restricted to A1, and knockdown of *Tc-kni* had no effect on abdominal segment identity, we found that combinatorial knockdown of two or more of these genes generated a higher frequency of abdominal transformations than would be expected additively, often of greater severity than those observed in single knockdowns (Fig. 4; Table S2).

Knocking down all three genes together produced the most severe phenotypes. Ninety-four percent of cuticles developing from embryos injected with all three dsRNAs formed jointed clawed legs on at least one abdominal segment (Fig. 4I,J, Table S2). These cuticles had an average of four extra pairs of partial or complete legs (not including the maxillary and labial legs induced by *Tc-gt* knockdown), and a maximum of seven extra pairs (Table S2), indicating homeotic transformation of up to seven abdominal segments.

### *Tc-nub*, *Tc-kni* and *Tc-gt* do not appear to act redundantly to regulate segment formation or head patterning

In addition to homeotic transformations, *Tc-gt* knockdowns result in truncations of the posterior abdomen with a very high penetrance (Fig. S7, Table S3; Bucher and Klingler, 2004). Knocking down *Tc-kni* and/or *Tc-nub* in addition to *Tc-gt* did not increase the penetrance or severity of these embryonic truncations (Fig. S7). Moreover, the frequency of truncations observed after eRNAi against *Tc-nub* + *Tc-kni* did not differ significantly from *GFP* controls (Fig. S7). These data suggest that the truncations observed after knockdown of *Tc-nub* *+* *Tc-gt* or knockdown of *Tc-nub* *+* *Tc-gt* *+* *Tc-kni* result primarily from loss of *Tc-gt*, and that neither *Tc-nub* nor *Tc-kni* plays any substantial role in segment addition. One caveat to this conclusion is that a higher proportion of triple knockdown embryos died before forming cuticle, when compared with double knockdowns (Table S2), and we observed that many triple knockdown embryos displayed severely disrupted patterning of *Tc-wg* stripes (e.g. see Fig. 6). Therefore, it may be that functional reduction/removal of all three genes has severe effects on the process of segment addition, or on other aspects of embryonic growth, that are masked by embryonic death.

*Tc-gt*, *Tc-nub* and *Tc-kni* are also co-expressed during head patterning. However, knocking down two or all three of these genes in parallel did not increase the penetrance or severity of head phenotypes – rather, knockdown effects were additive (Fig. S8), as might be expected if all three genes act independently.

### *Tc-nub*, *Tc-gt* and *Tc-kni* affect segment identity via Hox gene regulation

Development of partial or complete legs on abdominal segments has also been observed in double knockdowns of two abdominal Hox genes, *Tc-abd-A* and *Tc-Ultrabithorax* (*Tc-Ubx*, also known as *Tc-Ultrathorax*; Bennett et al., 1999; Lewis et al., 2000). To determine whether these Hox genes are misexpressed after eRNAi against *Tc-nub*, *Tc-kni* and *Tc-gt*, we performed HCR *in situ* hybridisation in embryos midway through segment addition (16-17 h AEL). This time point is shortly after the period during which *Tc-nub*, *Tc-gt* and *Tc-kni* are co-expressed, and should, therefore, reveal the immediate effects of knockdown on Hox gene expression.

**Fig. 5.**
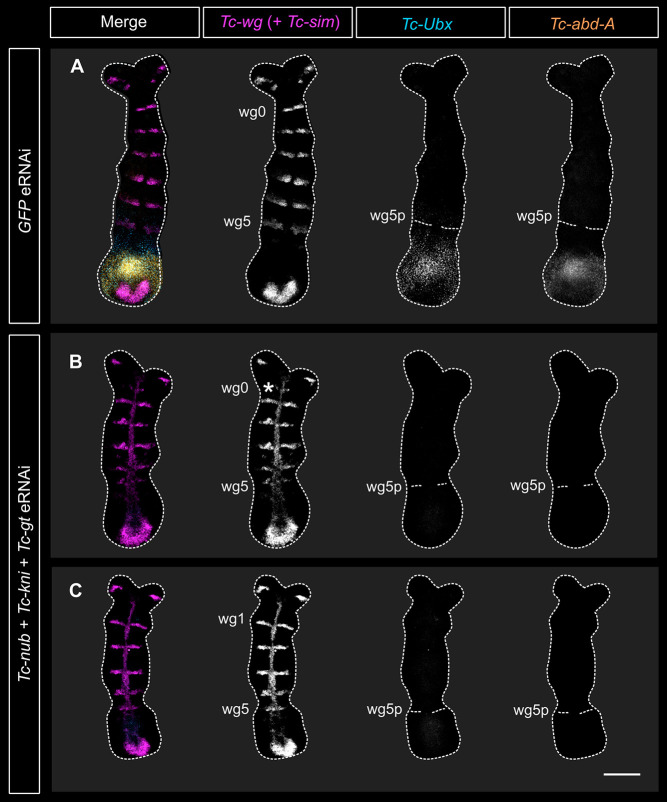
**Triple knockdown of *Tc-nub*, *Tc-gt* and *Tc-kni* expression eliminated *Tc-Ubx* and *Tc-abdA* expression in the SAZ.** (A) Embryos injected with *GFP* dsRNA (2 μg/μl) expressed *Tc-Ubx* and *Tc-abdA* in the SAZ (7/7 examined). This same embryo is presented in Fig. 6A. (B,C) At similar stages of segment addition, embryos injected with *Tc-nub*, *Tc-kni* and *Tc-gt* dsRNA (1 μg/μl each) did not express *Tc-Ubx* or *Tc-abdA* in the SAZ (3/3 examined). An asterisk marks the deteriorating, presumptive mandibular *Tc-wg* stripe (wg0) in B, which is likely deleted entirely in the embryo shown in C. Triple eRNAi embryos were also stained for the expression of the midline marker *Tc-single-minded* in this experiment, in the same channel as *Tc-wg*. All embryos were imaged using the same laser settings and brightness/contrast values were adjusted identically for all images. In all panels, anterior is to the top and ventral is along the vertical midline. wg0-5,*Tc-wg* stripes 0-5; wg0-5p, posterior boundary of wg0-5. Scale bar: 100 μm.

In wild-type embryos, expression of *Tc-Ubx* and *Tc-abdA* is detectable in the SAZ after the formation of the wg2 and wg4 stripes, respectively (Bennett et al., 1999; Shippy et al., 1998). Accordingly, we observed strong expression of both genes in the SAZ of control embryos (injected with GFP dsRNA) immediately after the formation of wg6 (Fig. 5A). In contrast, similarly staged embryos injected with *Tc-nub*, *Tc-kni* and *Tc-gt* dsRNA did not express *Tc-Ubx* or *Tc-abdA* (Fig. 5B). This loss of Hox gene expression is consistent with the dramatic abdominal phenotypes observed after triple eRNAi. The antennal and mandibular *Tc-Wg* stripes (wg0 and wg1) were deleted or highly disorganised in triple knockdowns, consistent with the head cuticle phenotypes observed in Fig. S7.

### *Tc-nub*, *Tc-gt* and *Tc-kni* regulate the expression of *Tc-Kr*, but not *Tc-hb*

We hypothesised that the repression of abdominal Hox genes observed after triple eRNAi might result from misregulation and expansion of other gap genes. The anterior borders of *Dm-Ubx* and *Dm-abd-A* expression in *Drosophila* are set primarily via direct repression by *Dm-hb* and *Dm-Kr*, respectively (Casares and Sánchez-Herrero, 1995; White and Lehmann, 1986). Therefore, we used HCR *in situ* hybridisation to examine the expression of both *Tc-hb* and *Tc-Kr* in embryos fixed at 16-17 h AEL following eRNAi against *Tc-nub*, *Tc-gt* and *Tc-kni*.

**Fig. 6.**
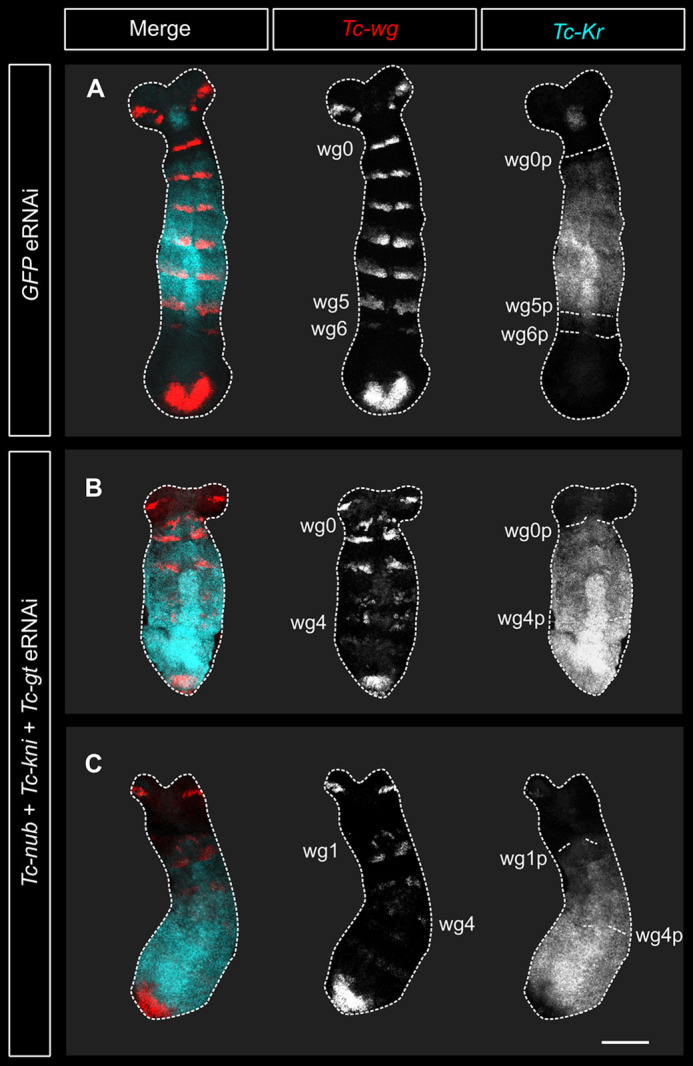
**Expression of *Tc-Kr* is expanded posteriorly after knocking down *Tc-nub*, *Tc-gt* and *Tc-kni*.** (A) In embryos injected with *GFP* dsRNA (2 μg/μl), *Tc-Kr* expression retracted from the SAZ to cover the presumptive thoracic segments (4/4 examined). This same embryo is presented in Fig. 5A. (B,C) In embryos injected with *Tc-nub*, *Tc-kni* and *Tc-gt* dsRNA (1 μg/μl each), *Tc-Kr* failed to retract from the SAZ (6/8 examined). The segmental expression of *Tc-wg* was extensively disrupted in the triple knockdown embryos displayed in this figure. The mandibular stripe (wg0) appears to be intact in B, but deleted in C (based on the spacing of stripes relative to the ocular *Tc-wg* stripes in the head). All embryos were imaged using the same laser settings and brightness/contrast values were adjusted identically for all images. In all panels, anterior is to the top and ventral is along the vertical midline. wg0-6, *Tc-wg* stripes 0-6; wg0-6p, posterior boundary of wg0-6. Scale bar: 100 μm.

We observed alterations in the pattern of *Tc-Kr*, but not *Tc-hb*, expression in embryos after simultaneous knockdown of *Tc-nub*, *Tc-gt* and *Tc-kni* (Fig. 6; Fig. S9). In wild-type embryos, *Tc-Kr* is expressed throughout the SAZ at the blastoderm stage, but becomes cleared from the posterior half of the SAZ during early germband formation (Cerny et al., 2008). This means that the SAZ is largely cleared of *Tc-Kr* expression by the time that the second trunk *Tc-wg* stripe (wg1) is formed (Tidswell, 2020). In contrast, triple-knockdown embryos with as many as four *Tc-wg* stripes showed little or no clearing of *Tc-Kr* expression in the SAZ (Fig. 6B,C). This means that after triple eRNAi, *Tc-Kr*, but not *Tc-hb*, is ectopically expressed in the SAZ.

Together, these data suggest that *Tc-nub*, *Tc-gt* and *Tc-kni* redundantly repress *Tc-Kr* expression, and that, in their absence, *Tc-Kr* expression expands into the abdominal primordia. We propose that this expansion leads to the repression of abdominal Hox genes, and subsequently to abdominal segment transformations.

### *Tc-nub* and *Tc-cas* play redundant roles in limb, but not segment, patterning

In addition to double and triple knockdowns of *Tc-nub* with *Tc-gt* and/or *Tc-kni*, we also performed simultaneous knockdown of *Tc-nub* and *Tc-cas* to determine whether they might play a redundant role in the posterior abdomen. Double *Tc-nub* *+* *Tc-cas* knockdowns do not display any posterior abdominal phenotypes, but 10/19 (52%) of cuticles examined exhibited defects in leg morphology. Specifically, the pretarsi, or claws, of the thoracic legs were almost entirely abolished (Fig. S10A-C). *Tc-nub* is expressed in the leg joints (Fig. S10D), as has been observed in other insect species (Li and Popadić, 2004; Turchyn et al., 2011). We observed that *Tc-cas* is also expressed in the developing legs, at both the proximal and distal ends (Fig. S10D). This is, to our knowledge, the first evidence suggesting that *cas* functions in arthropod limb development.

## DISCUSSION

In this study, we have shown that the genes *hb*, *Kr*, *nub* and *cas* are expressed sequentially in the SAZ of *Tribolium*, as they are in *Drosophila* neuroblasts. We have also shown that *Tc*-Nub plays a role in axial patterning, acting redundantly with the abdominal gap proteins *Tc*-Gt and *Tc*-Kni to repress *Tc-Kr* expression, and thereby to establish normal abdominal Hox gene expression. Our findings provide support for the theory that the neuroblast timer network was co-opted for axial patterning.

### Nub represses *Kr* expression redundantly with the gap and gap-like proteins Gt and Kni

Our combinatorial knockdown experiments indicate that *Tc*-Nub, *Tc*-Gt and *Tc*-Kni all contribute to the repression of *Tc-Kr* in the abdomen. *Dm*-Gt and *Dm*-Kni are known to repress *Dm-Kr* expression in *Drosophila* (Jaeger, 2011), and *Tc*-Gt has long been suspected to regulate *Tc-Kr* expression in *Tribolium* (Bucher and Klingler, 2004; Cerny et al., 2005). However, this is, to our knowledge, the first evidence that Kni regulates *Kr* expression in a non-drosophilid insect (Jaeger, 2011) and that Nub can repress *Kr* in the context of arthropod segment patterning.

*Tc-nub*, *Tc-kni* and *Tc-gt* seem to display ‘distributed redundancy’, i.e. they have different but overlapping roles, so that if one gene is lost, the others can at least partially compensate for it (Wagner, 2005). There are obvious reasons why the gap gene network might benefit from being robust to mutation. These genes regulate some of the earliest and most crucial elements of the insect body plan (segment boundaries and segment identities), and complete disruption of gap gene function is lethal (Jürgens et al., 1984; Nüsslein-Volhard et al., 1984; Wieschaus et al., 1984). The overlapping functions of *Tc-nub*, *Tc-gt* and *Tc-kni* may also be important for fine-tuning the expression dynamics of *Tc-Kr*, allowing for more precise regulation of the overlapping Hox gene domains in the posterior thorax and anterior abdomen.

Nub may also regulate *Kr* expression during axial patterning in other insect species, with varying degrees of redundancy with Gt and/or Kni*.* In *Oncopeltus*, pRNAi against *Oc-nub* results in prominent abdominal segment transformations arising from downregulation of *Oc-abd-A* expression (Hrycaj et al., 2008). We have shown that similar phenotypes arise in *Tribolium* from ectopic expression of *Kr.* Knockdown of *Oc-gt* or *Oc-kni* has no obvious effect on *Oc-Kr* expression (Ben-David and Chipman, 2010), suggesting that Nub may play a more central role than Gt and Kni in regulating *Kr* expression in *Oncopeltus*. In contrast, deletion of both *Drosophila nubbin* paralogues has little effect on the gap domain of *Dm-Kr* expression (Cockerill et al., 1993), despite the fact that Dm-Nub is able to repress *Dm-Kr* in neuroblasts (Grosskortenhaus, 2006; Tran and Doe, 2008). In this species, Gt and Kni may therefore have a more prominent role in *Kr* regulation than Nub. Intriguingly, we have observed subtle misexpression of *Dm-abd-A* expression in *Drosophila* embryos lacking both *nubbin* paralogues (Tidswell, 2020), in contrast to previous reports (Hrycaj et al., 2008). It therefore seems likely that Dm-Nub is able to repress *Dm-Kr* expression in the context of the gap gene network, but that this interaction is weak and/or masked by redundancy with Dm-Gt and Dm-Kni.

Subtle alterations in network interactions, even while the overall output of the network is conserved (known as developmental systems drift), are a common feature of the gap gene network (Crombach et al., 2016; Wunderlich et al., 2015). Investigating the functional overlap between Nub, Gt and Kni in additional insect species, with different modes of segmentation, and at strategic points in the insect phylogeny, will help to determine when and how the function of these genes has drifted over evolutionary time. This represents a promising framework for studying gene regulatory network evolution.

It is striking that the phenotypes observed after simultaneous knockdown of *Tc-nub*, *Tc-kni* and *Tc-gt* are very reminiscent of those observed after knockdown of the gap gene *Tc-mille-pattes* (*Tc-mlpt*). Both treatments lead to the expansion of *Tc-Kr* expression into the SAZ, and the formation of ectopic legs on presumptive abdominal segments (Savard et al., 2006). It may be that Tc-Mlpt is required for the expression not just of *Tc-gt* (Savard et al., 2006), but also of *Tc-kni* and *Tc-nub*, in the SAZ. Knockdown of *Tc-mlpt* expression would then effectively phenocopy a triple knockdown of *Tc-nub*, *Tc-gt* and *Tc-kni* in the SAZ. This hypothesis places *Tc-mlpt* in a crucial position in the gap gene network and warrants further investigation.

### A central role for the neuroblast timer genes for Hox gene regulation in *Tribolium*?

**Fig. 7.**
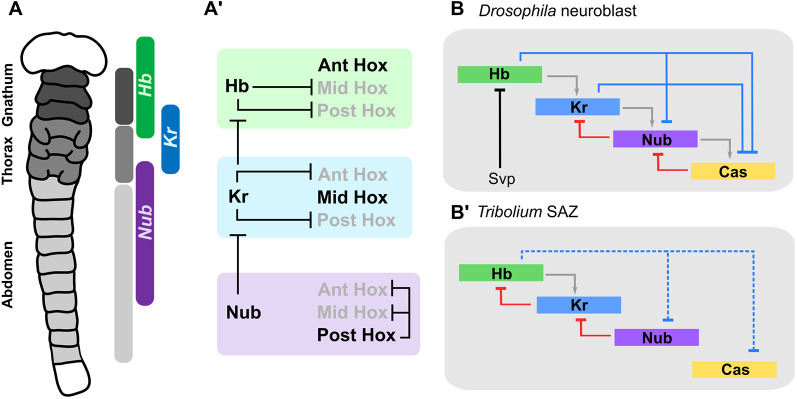
**Hox and cross-regulation by the neuroblast timer proteins.** (A) Expression of the first three genes of the neuroblast timer series broadly aligns with the three trunk tagma in *Tribolium*. (A′) Interactions between *Tc-hb*, *Tc-Kr* and *Tc-nub*, and the Hox genes are theoretically sufficient to generate three distinct domains of Hox gene expression, broadly aligning with the three major body tagma. Ant, anterior; Mid, middle; Post, posterior. (B,B′) Summary of known or predicted interactions between the neuroblast timer genes in *Drosophila* neuroblasts (B) and in the SAZ of *Tribolium* (B′). Interactions presented in B are based on published models (Averbukh et al., 2018; Nakajima et al., 2010). Svp, the nuclear transcription factor Seven-up (Kanai et al., 2005). Interactions presented in B′ are based on data from Marques-Souza et al. (2008) and this article. Interactions are colour-coded to represent four major ‘classes’ of interaction thought to contribute to sequential expression of the neuroblast timer genes: red, feedback repression; blue, ‘next-plus-one’ repression; grey, feed-forward activation; black, external inputs. At least in the neuroblast timer network, repression between network components seems to be more significant for network dynamics than activation (Averbukh et al., 2018). We infer repression of *Tc-nub* and *Tc-cas* by *Tc*-Hb as likely based on their mutually exclusive expression domains in the abdomen (Fig. 2), but these interactions are indicated using dotted lines in B′ to signify that they are yet to be experimentally demonstrated.

It is intriguing to note that the expression domains of the first three neuroblast timer genes, *Tc-hb*, *Tc-Kr* and *Tc-nub*, align approximately with the three trunk tagma in *Tribolium* (gnathum, thorax and abdomen, respectively), save that they are shifted anteriorly to align with parasegment boundaries, and *Tc-nub* covers most but not all of the abdominal parasegments (Fig. 7A).

Functional data also support the importance of this gene-tagma pattern (Fig. 7A′). *Tc*-Hb represses thoracic and abdominal Hox genes (Marques-Souza et al., 2008), allowing gnathal Hox genes to be expressed. *Tc*-Kr represses gnathal (Cerny et al., 2005) and abdominal Hox genes, allowing the thoracic Hox genes to be expressed. Finally, *Tc*-Nub, in tandem with *Tc*-Gt and *Tc*-Kni, represses *Tc-Kr* expression, which, in the absence of *Tc*-Hb, allows for abdominal Hox genes to become expressed. This minimal network could therefore provide enough information to lay down the basic functional divisions of the insect axis (although not, of course, the fine details of individual segment identity).

This observation is particularly intriguing because gap gene regulation of Hox genes is thought to pre-date gap gene regulation of segment boundary positions (Clark et al., 2019). However, we lack a detailed understanding of how the gap gene network as a whole contributes to Hox gene regulation in sequentially segmenting insects such as *Tribolium*. A comprehensive molecular dissection of Hox gene regulation in *Tribolium* is required to test this hypothesis.

### Co-option of the neuroblast timer series for axial patterning in insects

The idea that the neuroblast timer network might be used for axial patterning in insects was first suggested when *Dm-hb*, *Dm-Kr*, *Dm-nub* and *Dm-cas* were found to be expressed, in that order, along the AP axis of the *Drosophila* embryo (Isshiki et al., 2001). We have shown that the genes of the neuroblast timer network are also expressed during axial patterning in the sequentially segmenting insect *Tribolium*, and that *Tc-nub* has a clear function during this process.

The roles of *hb*, *nub* and *cas* in the neuroblast timer network long predate their roles in axial patterning. Homologues of all three genes (*Ikaros*, *PouF2* and *Casz1*, respectively) are expressed sequentially in neural and/or retinal stem cells in mammals and promote the formation of a temporal sequence of different daughter cell types (Alsiö et al., 2013; Elliott et al., 2008; Javed et al., 2020; Mattar and Cayouette, 2015; Mattar et al., 2015). In contrast, there is no evidence that any of these genes play a role in segment formation or axial patterning outside of the arthropods. Even within the non-insect arthropods, there are species that express *hb* and/or *Kr* in their neuroblasts but not in the SAZ (Chipman and Stollewerk, 2006; Kontarakis et al., 2006). From these observations, we can infer that at least *hb*, *nub* and *cas* were most likely recruited to a role in axial patterning from an ancestral role in neural patterning.

Beyond the broad similarities presented in this paper, we have also identified some key differences in the order of expression and function of neuroblast timer genes in neuroblasts and in the SAZ. First, *hb* is expressed in the SAZ after *cas*, something that is not observed in neuroblasts. This posterior domain of *hb* is conserved in a range of insect lineages (Jaeger, 2011; Liu and Kaufman, 2004a; Marques-Souza et al., 2008; Mito et al., 2005), and has been hypothesised to influence the duration of segmentation (Nakao, 2016). It seems likely, then, that it is a significant component of the timer network in the SAZ. Furthermore, although we found a clear role for *nub* in regulating axial identities, we have found no such role for *cas*. Unlike the other neuroblast timer genes, expression of *cas* in the SAZ of *Tribolium* is modulated in a complex pair-rule pattern, arguing against its regulating axial identity across a broad, continuous region of the SAZ. The function of the ‘gap-like’ domain of *cas* expression in *Drosophila* also remains mysterious, as *Dm-cas* mutants appear normal outside their neural defects (Mellerick et al., 1992). It may be that *cas*, like *nub*, acts redundantly with other genes to exert its influence on axial identity; that it has lost the ability to regulate axial identity in *Tribolium* and *Drosophila*; or that it never had such a role. Analysis of *cas* expression and function in the SAZs of other insect species may help to distinguish between these possibilities.

It is worth asking whether the regulatory interactions that drive sequential expression of *Tc-hb*, *Tc-Kr*, *Tc-nub* and *Tc-cas* in the SAZ are the same as in *Drosophila* neuroblasts. Sequential expression of the neuroblast timer genes in *Drosophila* neuroblasts depends largely on cross-regulatory interactions between their gene products, including feed-forward activation, feedback repression and ‘next-plus-one’ repression (Averbukh et al., 2018; Doe, 2017; Isshiki et al., 2001; Nakajima et al., 2010; Rossi et al., 2017) (Fig. 7B). Our understanding of *Tribolium* gap gene interactions is fragmentary, but this network may share some of its regulatory interactions with neuroblasts (Fig. 7B′).

However, there are also obvious differences between the two networks. The transition between *Tc-hb* and *Tc-Kr* expression in the SAZ appears to be mediated entirely by interactions within the network (Marques-Souza et al., 2008), while in neuroblasts, the transition between *Dm-hb* and *Dm-Kr* expression is driven by the nuclear receptor *Dm*-Seven-Up in a cytokinesis-dependent manner (Benito-Sipos et al., 2011; Grosskortenhaus et al., 2005; Kanai et al., 2005; Mettler et al., 2006). Furthermore, the timing and extent of *Tc-Kr* expression in the SAZ is influenced by gap genes that are not expressed in neuroblasts, such as *Tc-gt* and *Tc-kni* (Bucher and Klingler, 2004; Cerny et al., 2008; this article).

By demonstrating sequential expression of the neuroblast timer genes in the SAZ of *Tribolium*, and revealing that *Tc-nub* is able to repress the expression of *Tc-Kr* to influence Hox gene expression, our findings provide strong support for the hypothesis that the neuroblast timer network has been co-opted for axial patterning during the evolution of insects. These findings will provide a basis for future studies examining the evolution and structure of the gap gene network in insects.

## MATERIALS AND METHODS

### *Tribolium castaneum* husbandry

*Tribolium castaneum* strain San Bernadino beetles (provided by A. Peel, University of Leeds, UK) were reared on organic wholemeal flour (Doves Farm Foods, Hungerford, UK) supplemented with fast action dried yeast (Sainsbury's, London, UK) and the antifungal agent Fumagilin-B (Medivet) at 30°C, as described in the Beetle Book v1.2 (Bucher, 2009). Egg lays were performed on strong white organic bread flour (Doves Farm Foods, Hungerford, UK). Incubators were maintained between 40-60% relative humidity where possible, and no day/night cycle was used (beetles were kept in the dark).

### Collection and fixation of wild-type embryos

*Tribolium* were allowed to lay on white flour for 24 h and their eggs were then collected using a sieve with a 200 μM mesh size (Retsch test sieve 200 mm×50 mm). Collected eggs were transferred into small mesh baskets (with a mesh aperture of 250 μm) and were rinsed several times in double-distilled H_2_O to remove all traces of flour. Their chorions were then removed by washing twice in bleach diluted with double-distilled H_2_O to a final concentration of 2.5% (v/v) hypochlorite, for 30-45 s. After further rinsing in double-distilled H_2_O, dechorionated embryos were fixed as described by Marques-Souza et al. (2008), except a 0.68 mm ID (internal diameter) needle was used to enhance devitellinisation rather than a 0.9 mm ID needle. Fixed and devitellinised embryos were stored in 100% methanol at −20°C.

### Ovary dissection and fixation

Ovaries were removed from adult female beetles in PBS using forceps. Dissected ovaries were transferred directly into 4% formaldehyde in PBT (PBS+0.01% Tween) on ice. An equal volume of heptane was added, and the tubes then rocked on a nutator for 20 min to allow for fixation. The ovaries were then rinsed several times in PBT and then washed into 100% methanol for storage at −20°C.

### RNA interference

Plasmids containing clones for *GFP*, *Tc-nub*, *Tc-cas*, *Tc-gt*, *Tc-kni* and *Tc-odd* were provided by A. Peel (University of Leeds, UK) and R. Sharma (University of Cambridge, UK) (clone sequences provided in Table S4). All dsRNA fragments used were computationally predicted to have a low potential for off-target gene silencing using the default search parameters of Deqor version 3.0 (i.e. the quality score of all potentially cross-silencing siRNAs was >5) (Henschel et al., 2004). dsRNA was synthesised from PCR products using T7 polymerase, and was purified using phenol chloroform precipitation. Purified dsRNA was resuspended in RNase-free water and injected into *Tribolium* adults or eggs at a concentration of 1-4 μg/μl. Unless specified otherwise, single knockdowns were carried out using 2 μg/μl of dsRNA, while double and triple knockdowns used the component dsRNAs mixed to a final concentration of 1 μg/μl each (the viscosity of the injection fluid became difficult to work with above 4 μg/μl).

All injections for RNAi were carried out using a Pico-injector system (Medical Systems). Parental RNAi was carried out by injecting dsRNA into the dorsal surface of the abdomen under the elytra of adult female beetles as described by Posnien et al. (2009). Males were introduced to the injected females the day after injection, and eggs were collected starting from 1 week after injection. Eggs were collected and fixed regularly (every 1-2 days) as described above for 3-4 weeks after injection.

Embryonic microinjection for eRNAi was carried out using a method adapted from Benton (2018). One- to 2-h-old eggs were transferred into small mesh baskets (with a mesh aperture of 250 μm) and rinsed several times in double-distilled H_2_O. Chorions were removed by washing twice in bleach, diluted with double-distilled H_2_O to a final concentration of ∼0.6% (v/v) hypochlorite, for 30-45 s. Eggs were rinsed again and then healthy looking eggs were lined up on coverslips and allowed to dry. Eggs were covered with a 1:1 mix of Halocarbon oil 700 and Halocarbon oil 27 (Sigma Aldrich) and dsRNA was injected into the anterior pole (to reduce the risk of damage to the posterior segment addition zone). The coverslip was turned over on to a Lumox culture dish as described by Benton (2018), except that glass ‘feet’ ∼0.6 mm high (made from strips of #1.5 coverslip) were attached to the coverslip at either end of the injected rows of eggs, to prevent them from being pressed against the membrane. Injected eggs were then stored in plastic chambers with wet paper towel (to maintain humidity) and reared at 30°C.

For fixation, injected embryos were aged for the appropriate length of time then injected with PBT+10% formaldehyde (v/v) and left to fix at room temperature for 1 h. They were then transferred using an eyelash hair to Eppendorf tubes and fixed for an additional hour in a 1:1 mix of heptane and PBT+4% formaldehyde (v/v). The aqueous layer was removed and 100% ice-cold methanol added. Germbands were manually dissected away from the remainder of the yolk, chorion and vitelline membrane in PBS, and then stored in 100% methanol at −20°C until required.

### Hybridisation chain reaction *in situ* hybridisation

Version 3.0 HCR probes (20 pairs per gene) and fluorescently labelled hairpins were produced by Molecular Instruments. Probe template sequences were taken from NCBI (*Tc-hb*, NM_001044628.1; *Tc-Kr*, NM_001039438.2; *Tc-nub*, XM_015979462.1; *Tc-cas*, XM_015980923.1; *Tc-wg*, NM_001114350.1; *Tc-Ubx*, XM_008203013.2; *Tc-abd-A*, NM_001039429.1). All required buffers were made according to the instructions provided by Molecular Instruments, with the one exception that the percentage of dextran sulphate in the probe hybridisation and amplification buffers was reduced from 10% (v/v) to 5% (v/v) to reduce viscosity and improve retention of embryos during washes.

Fixed embryos or ovaries were prepared for hybridisation chain reaction (HCR) *in situ* hybridisation (ISH) by removing methanol and replacing it with 1 ml of PBT containing 4% formaldehyde. Tubes were rocked on the nutator for 30 min to allow for additional fixing and rehydration to occur. The HCR ISH was then carried out as per the Molecular Instruments HCR v3.0 protocol for whole-mount fruit fly embryos, with the exception that hybridisation steps were carried out in 100 rather than 200 μl of hybridisation buffer, and the volume of probe added was adjusted to give the same final concentration (4 nM). Additionally, 1 ng/μl DAPI was added to the first 30 min wash on the final day so that nuclear staining could be carried out in parallel. After washing, embryos or ovaries were transferred first into 25% (v/v) glycerol and then into 50% (v/v) glycerol before being stored at 4°C to stiffen and clear for mounting.

### Mounting and imaging of embryos and ovarioles

Blastoderm stage embryos were mounted in glass-bottomed Petri dishes (Cellvis), and dissected germbands and whole ovarioles on glass slides, in ProLong Gold Antifade Mountant (Thermofisher Scientific) as per the manufacturer's instructions. Most mounted embryos and ovarioles were imaged using an Olympus FV3000 confocal microscope and associated FLUOVIEW software at the Department of Zoology Imaging Facility (University of Cambridge). 12-bit *z*-stacks of entire embryos and ovarioles were taken using a UPLSAPO 20× objective lens (no immersion, NA=0.75) with a *z* step-size of 3-5 μM and a pixel dwell time of 2 μs. Z-stacks spanned the entire depth (from ventral to dorsal surface) of flat-mounted embryos, and approximately half of the depth of blastoderm-stage embryos. A minority of embryos (several pictured in Fig. 1 and all pictured in Fig. S1) were imaged prior to the installation of the Olympus FV3000 microscope, using a Leica SP5 inverted confocal microscope at the Department of Zoology Imaging Facility (University of Cambridge). 16 bit *z*-stacks of embryos pictured in Fig. 1 were taken using a 11506191 20× objective lens (no immersion, NA=0.7), with a *z* step-size of 1-3 μm. 16 bit *z*-stacks of the posterior gut regions pictured in Fig. S1 were taken using a 11506192 63× objective lens (oil immersion, NA=1.4), with a *z* step-size of 0.3-0.5 μM. All images were taken with a scan format of 1024×1024 pixels. A 405 laser was used to visualise DAPI, and 488, 561, 594 and 633 (Leica) or 640 (Olympus) lasers were used to visualise fluorescently tagged HCR ISH hairpins.

### Preparation and imaging of cuticles

Embryos and larvae were processed for cuticle preparation either upon hatching, or after 7-10 days if they failed to hatch in this time. Embryos and larvae of uninjected embryos were first rinsed in 2.5% (v/v) bleach and then in double-distilled H_2_O to remove any remaining chorion and debris. Injected embryos and larvae were dissected out of their chorions manually, and washed in methanol and then heptane (1 h each) to remove the halocarbon oil. Embryos or larvae were then transferred to a glass slide, covered with a 1:1 mix of Hoyer's medium (Dahmann, 2008):lactic acid and a coverslip, and heated at 60°C overnight. Cuticles were imaged with an excitation wavelength of 633 nm on an Olympus FV3000 confocal microscope in the Department of Zoology (University of Cambridge). Overview images of entire cuticles were taken using a UPLSAPO 10× objective lens (no immersion, NA=0.4) with a *z* step-size of 4-5 μm, while close-up images of cuticles (i.e. the inset in Fig. 4B, and the cuticle images in Figs S7 and S9) were taken using with a UPLSAPO 20× objective lens (no immersion, NA=0.75) with a *z* step-size of 1-2 μm. All images were taken as 12 bit, with a scan format of 1024×1024 pixels and a pixel dwell time of 2 μs.

### Image processing and figure assembly

Images and *z*-stacks were stitched using the Olympus FV3000 FLUOVIEW software. Additional image processing was carried out in Fiji (Schindelin et al., 2012). To correct for subtle misalignment of the dichroic mirrors on the confocal microscope used for imaging, channels of images taken using the Olympus FV3000 confocal microscope were realigned using the ‘Olympus FV3000 dichroic mirror offsets’ Fiji plug-in by Matthew Wayland (https://github.com/WaylandM/dichroic-mirror-offsets). Unless otherwise stated, all images of embryos are maximum projections of confocal *z*-stacks. Fiji was also used to adjust image brightness and contrast, and to rotate, crop and re-slice images where necessary, in accordance with guidelines presented by Schmied and Jambor (2021). Fixed intensity values were applied for qualitative comparisons of signal intensity between images. We used the ChrisLUTs LUT package for ImageJ (Christophe Leterrier and Scott Harden; https://github.com/cleterrier/ChrisLUTs) for presenting selected confocal images. Figures were assembled in the open source vector graphics editor Inkscape (https://inkscape.org/).

### Statistical analysis

Statistical analysis was carried out using R (v4.1.0) (R Core Team, 2021a) and RStudio (v1.4.1106) (R Core Team, 2021b). Bayesian logistic regressions were carried out using the *bayesglm* function in the *arm* (v1.11-2) package (Gelman and Su, 2020).

## Supplementary Material

Supplementary information

Reviewer comments
