# Supplementary material for: The neuroblast timer gene nubbin exhibits functional redundancy with gap genes to regulate segment identity in Tribolium
Source: Development. 2021 Aug 19;148(16):dev199719. doi: 10.1242/dev.199719 (PMC8406537; doi:10.1242/dev.199719)
Supplement: Supplementary information [file develop-148-199719-s1.pdf]

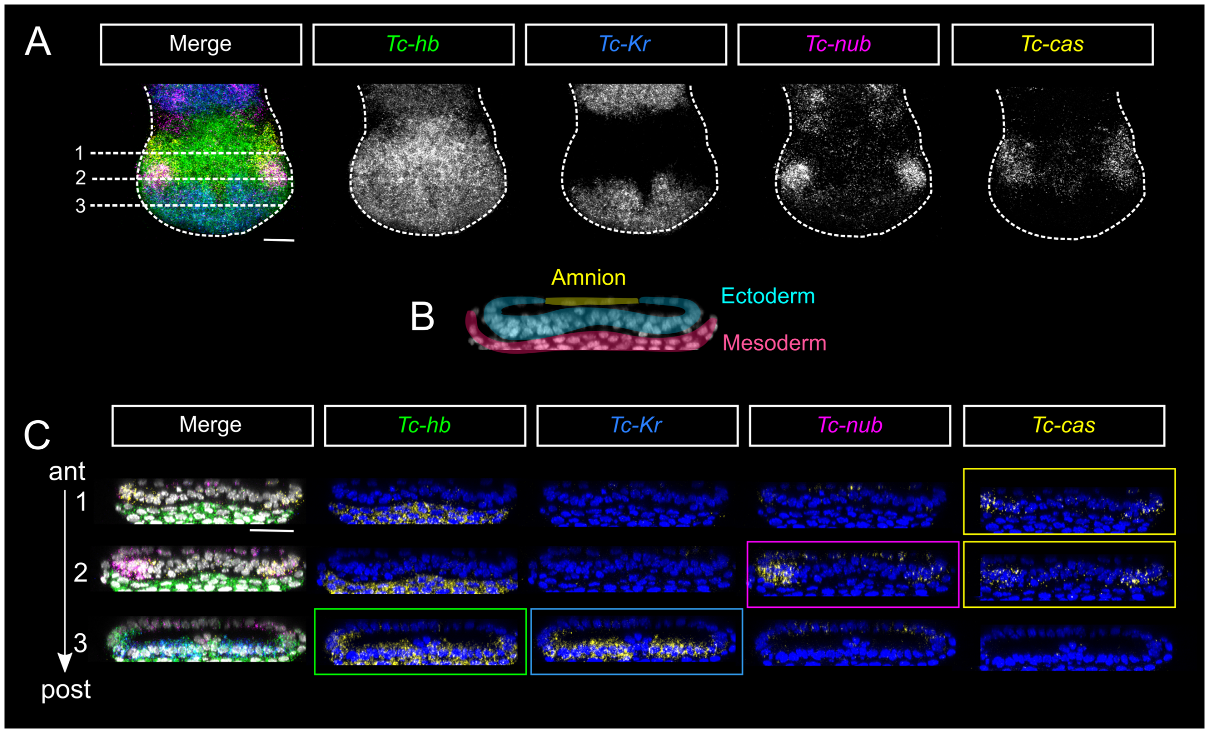

Fig. S1. *Tc-hb*, *Tc-Kr*, *Tc-nub* and *Tc-cas* are all expressed in the ectoderm at the posterior-most end of the embryo (the presumptive hindgut). A) *Tc-hb*, *Tc-Kr*, *Tc-nub* and *Tc-cas* are expressed in overlapping domains in posterior-most region of the embryo after segment addition is completed, but before gut morphogenesis begins. Anterior is to the top, and ventral is along the vertical midline. **B)** A transverse section of the posterior SAZ showing DAPI in grey and the arrangement of amnion, ectoderm and mesoderm (as judged by tissue morphology) in false colours. Dorsal is to the top. **C)** Transverse sections of the posterior SAZ from the same embryo shown in A) at three positions along the anterior-posterior axis (labelled as 1, 2 and 3). At position 1, the most anterior position, only *Tc-cas* is expressed in the ectoderm. At position 2, the central position, both *Tc-nub* and *Tc-cas* are expressed in the ectoderm. Finally, at position 3, the most posterior position, *Tc-hb* and *Tc-Kr* are expressed in the ectoderm. *Tc-hb* is expressed in the mesoderm throughout the SAZ. Dorsal is to the top. Each of the maximum projections in panels B and C spans approximately 5-10 µM along the anterior-to-posterior axis of the embryo. Scale bar = 20 µM.

Development • Supplementary information

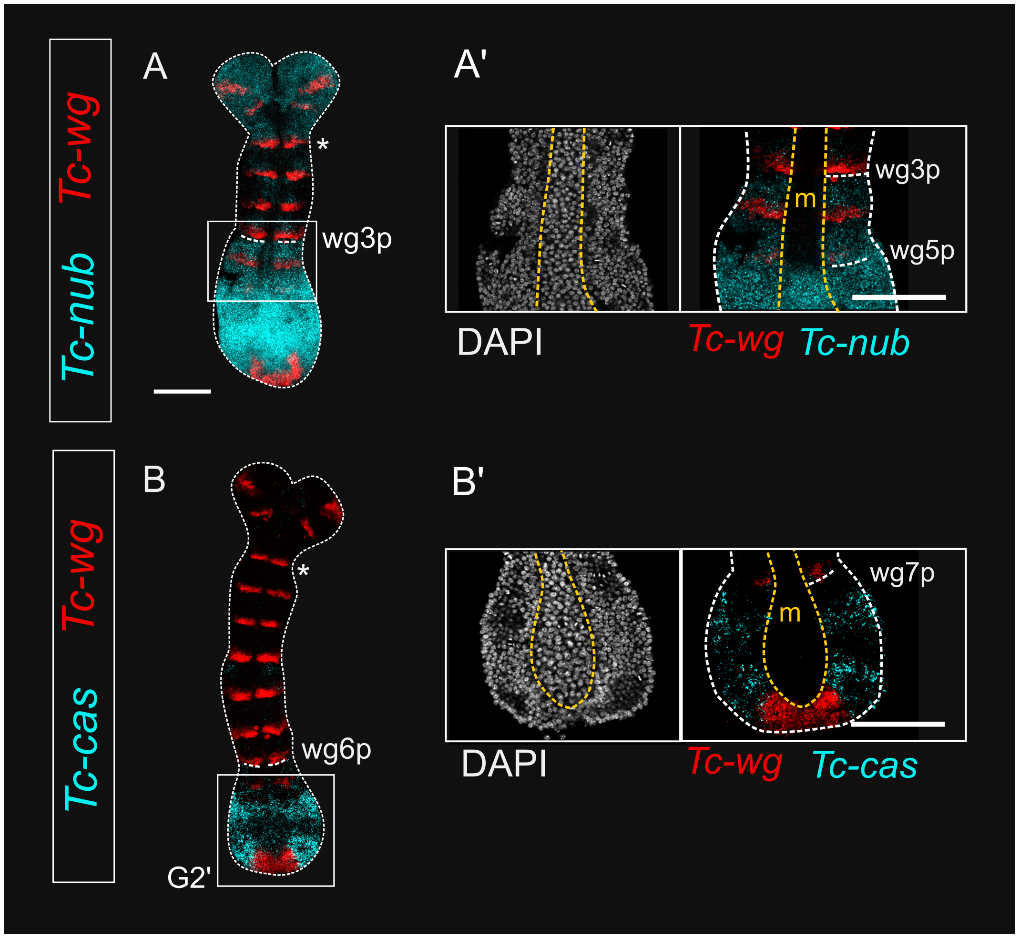

Fig. S2. *Tc-nub* and *Tc-cas* are expressed differently in the ectoderm and mesoderm of the SAZ. Panels A and B are maximum projections of confocal z-stacks through dissected, flat mounted embryos (the same embryos are shown in Fig 3), while panels A' and B' are single horizontal sections through the regions indicated in panels A and B. **A-A')** Just after the formation of wg5, the anterior boundary of *Tc-nub* expression abuts wg3p in the ectoderm (lateral), but wg5p in the mesoderm (m, outlined with yellow dotted lines). **B-B')** *Tc-cas* is expressed in the ectoderm, but not the mesoderm of the SAZ. In all panels, anterior is to the top and ventral is along the vertical midline of the embryo. Asterisks mark the first *Tc-wg* stripe to form in the trunk (wg0). wg3-7p = the posterior boundaries of wg3-7. Scale bar = 100 μM.

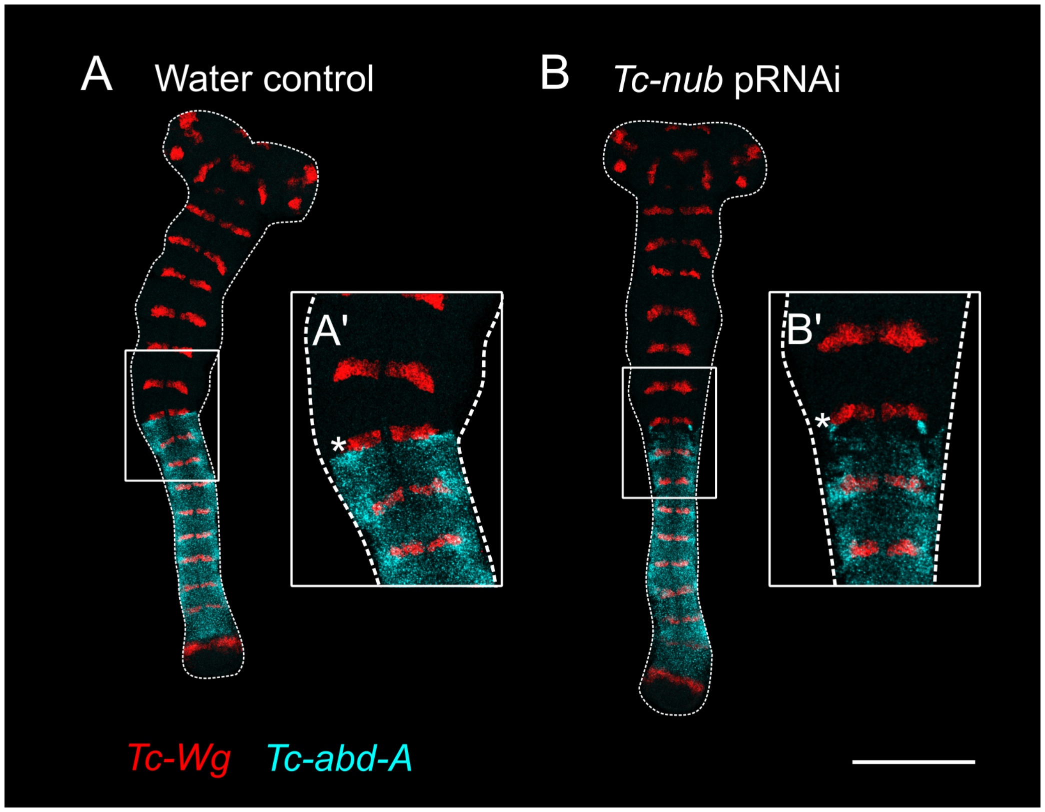

Fig. S3. Expression of the Hox gene *Tc-abd-A* in parasegment 7 was disrupted following *Tc-nub* pRNAi. (A) Embryos produced by mothers injected with water showed normal expression of *Tc-abd-A* in the anterior abdomen, abutting the posterior of wg6 (marked by an asterisk in the magnified inset A'). **(B)** Embryos produced by mothers injected with *Tc-nub* dsRNA (2 µg/µL) showed disrupted *Tc-abd-A* expression in the anterior of parasegment 7, just posterior to wg6 (marked with an asterisk in the magnified inset B'). Anterior is to the top and ventral is along the vertical midline of the embryo. Scale bar = 200 µM.

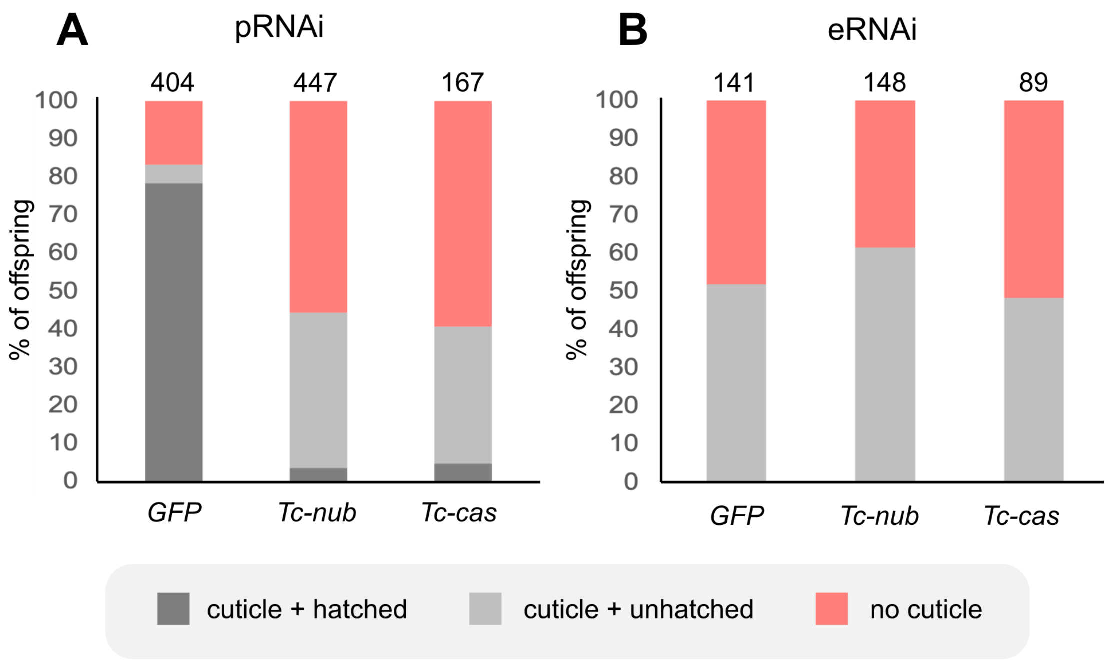

Fig. S4. *Tc-nub* and *Tc-cas* RNAi reduced the proportion of embryos developing to the point of cuticle formation and hatching compared to *GFP* controls. (A) pRNAi against *Tc-nub* or *Tc-cas* (2 µg/µL) reduced the percentage of eggs forming cuticle from ~80% (in *GFP* pRNAi controls) to less than 50%. Furthermore, many of the eggs that did form apparently normal cuticle after *Tc-nub* or *Tc-cas* pRNAi failed to hatch. Note that water and *GFP* controls gave similar results for pRNAi (Table S1). **(B)** After eRNAi, the percentage of eggs forming cuticle was similar in *GFP* controls compared to *Tc-nub* or *Tc-cas* knockdowns (all dsRNAs injected at 2 µg/µL). Hatching rates were not recorded for eRNAi as maintenance of embryos in halocarbon oil supressed hatching in all treatments. The number of beetles (A) or embryos injected (B) for each treatment is indicated above each bar. More details are available in Tables S1 and S2.

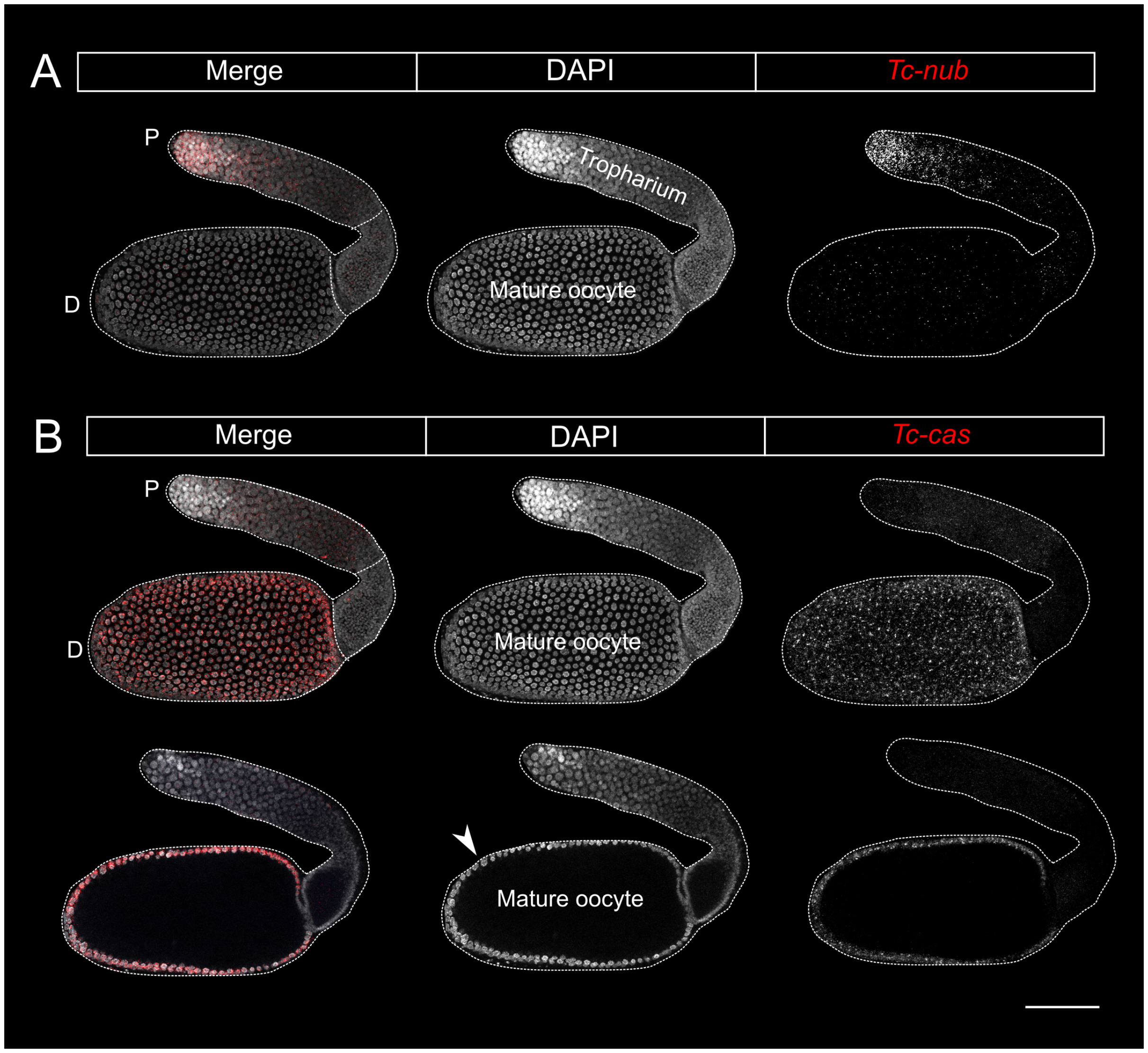

Fig. S5. *Tc-nub* and *Tc-cas* are expressed in the ovarioles of mature female *Tribolium*. (A) *Tc-nub* is expressed in a subset of nurse cells in the proximal tropharium. **(B)** *Tc-cas* is expressed in the follicular cells that surround the mature oocyte. The upper row of images show a maximum projection through an entire dissected ovariole, while the lower row of images show a maximum projection through ~10 µM of the ovariole's center, illustrating that *Tc-cas* expression is limited to the layer of follicular cells surrounding the oocyte (white arrowhead) but is absent from the oocyte itself. P = proximal end of the ovariole, D = distal end of the ovariole. Scale bar = 100 µM.

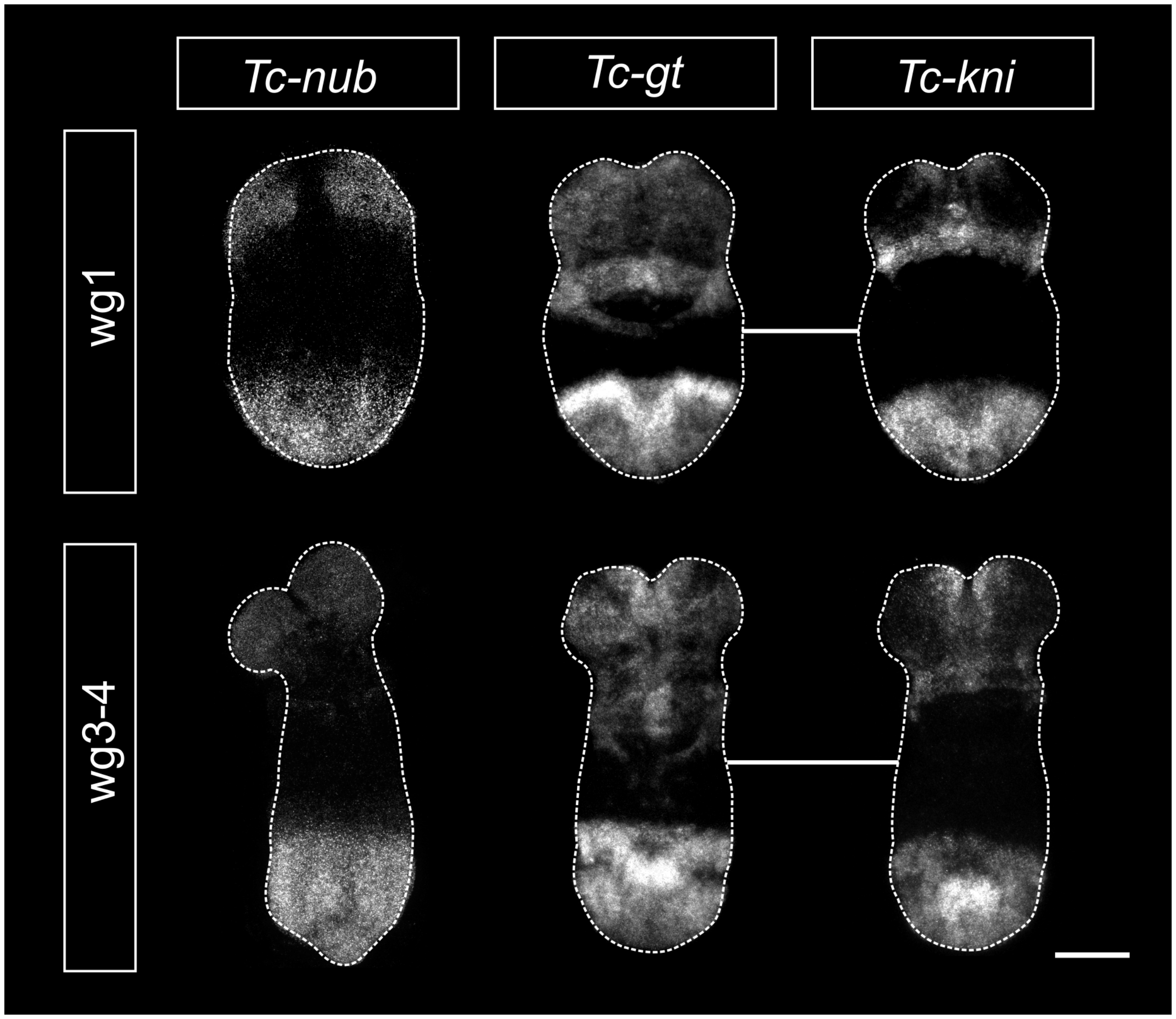

Fig. S6. *Tc-nub*, *Tc-gt* and *Tc-kni* have overlapping expression domains in the SAZ during early germband extension. The row headers indicate the identity of the most recently formed *Tc-wg* stripe as a proxy for developmental stage. *Tc-gt* and *Tc-kni* images were taken from the same embryo, indicated by a white line joining them. Images are maximum projections through flat mounted, dissected germbands. Scale bar = 100 µM.

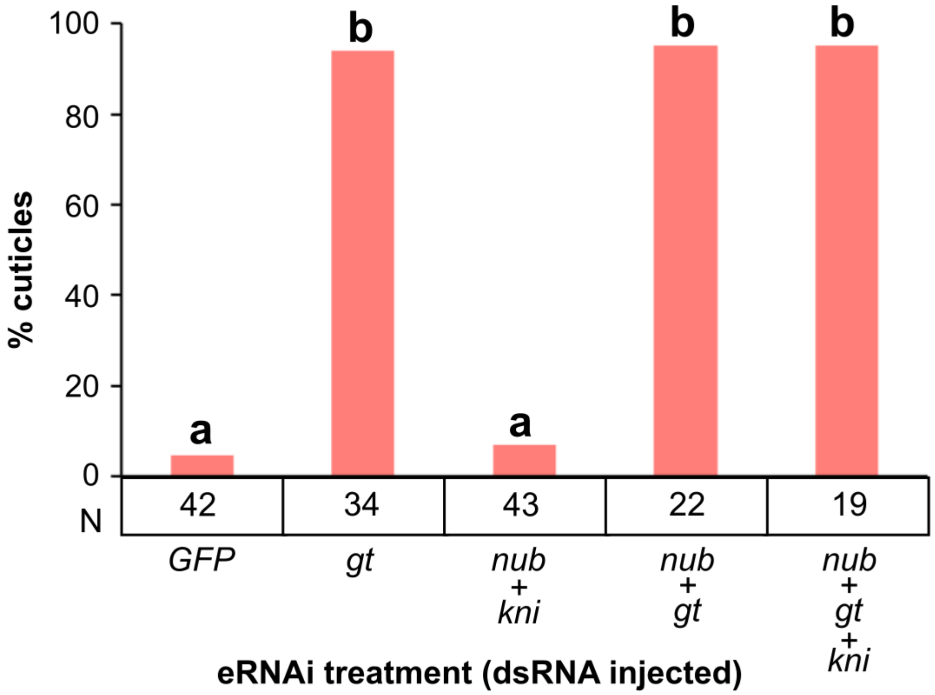

Fig. S7. Knockdown of *Tc-gt*, but not *Tc-nub* or *Tc-kni*, by eRNAi induced a high frequency of axial truncations. Single knockdowns were carried out using dsRNA at a concentration 2 μg/μL, while in double and triple knockdowns each component dsRNA was at a concentration of 1 μg/μL. A Bayesian logistic regression of axial truncation frequency on eRNAi treatment indicated that eRNAi treatments differed significantly in their odds of generating axial truncations (χ^2 (df=4) = 151.84, $p<2.2\text{x}10^{-6}$). A Tukey post-hoc test was used to determine significant differences between groups, indicated as the letters on top of each column; treatments marked with an 'a' are significantly different from those marked 'b' at the $p<1\text{x}10^{-5}$ level. The number of cuticles examined from each treatment is indicated in the row labelled 'N', below the X-axis.

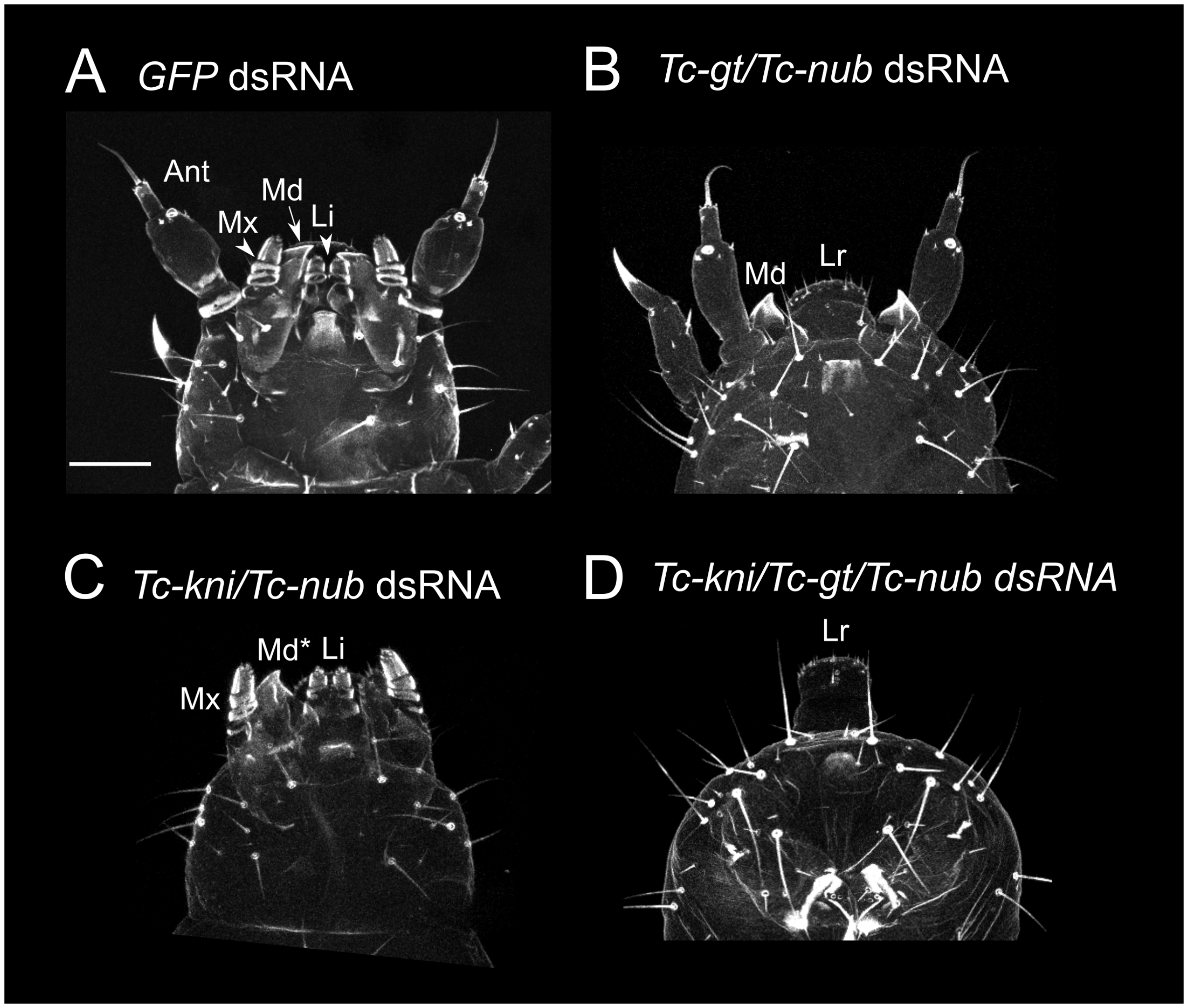

Fig. S8. *Tc-nub* eRNAi did not enhance the effects of *Tc-gt* or *Tc-kni* knockdown on external head development. (A) Embryos injected with *GFP* dsRNA (2 µg/µL) had wild type external head morphology. **(B)** In embryos injected with *Tc-nub* + *Tc-gt* dsRNA (1 µg/µL each), the maxillae and labium were transformed into legs, while the mandibles, antennae and labrum were left intact, as observed in *Tc-gt* single knockdowns. **(C)** In embryos injected with *Tc-kni* + *Tc-nub* dsRNA (1 µg/µL each), the antennae and one or more mandibles was lost, but the maxillae, labium and labrum remained intact, as observed in *Tc-kni* single knockdowns. (D) Embryos injected with *Tc-kni* + *Tc-gt* + *Tc-nub* dsRNA (1 µg/µL each) displayed an additive phenotype; the antennae and mandibles are lost, while the maxillae and labium are transformed into legs. These data suggest that *Tc-nub* does not act redundantly with *Tc-kni* and/or *Tc-gt* to regulate head development in *Tribolium*. An = antenna; Md = mandible; Mx = maxilla; Li = labium; Lr = labrum. In C, Md* indicates the single remaining mandible (the second mandible is lost in this individual). Scale bar is 50 µM.

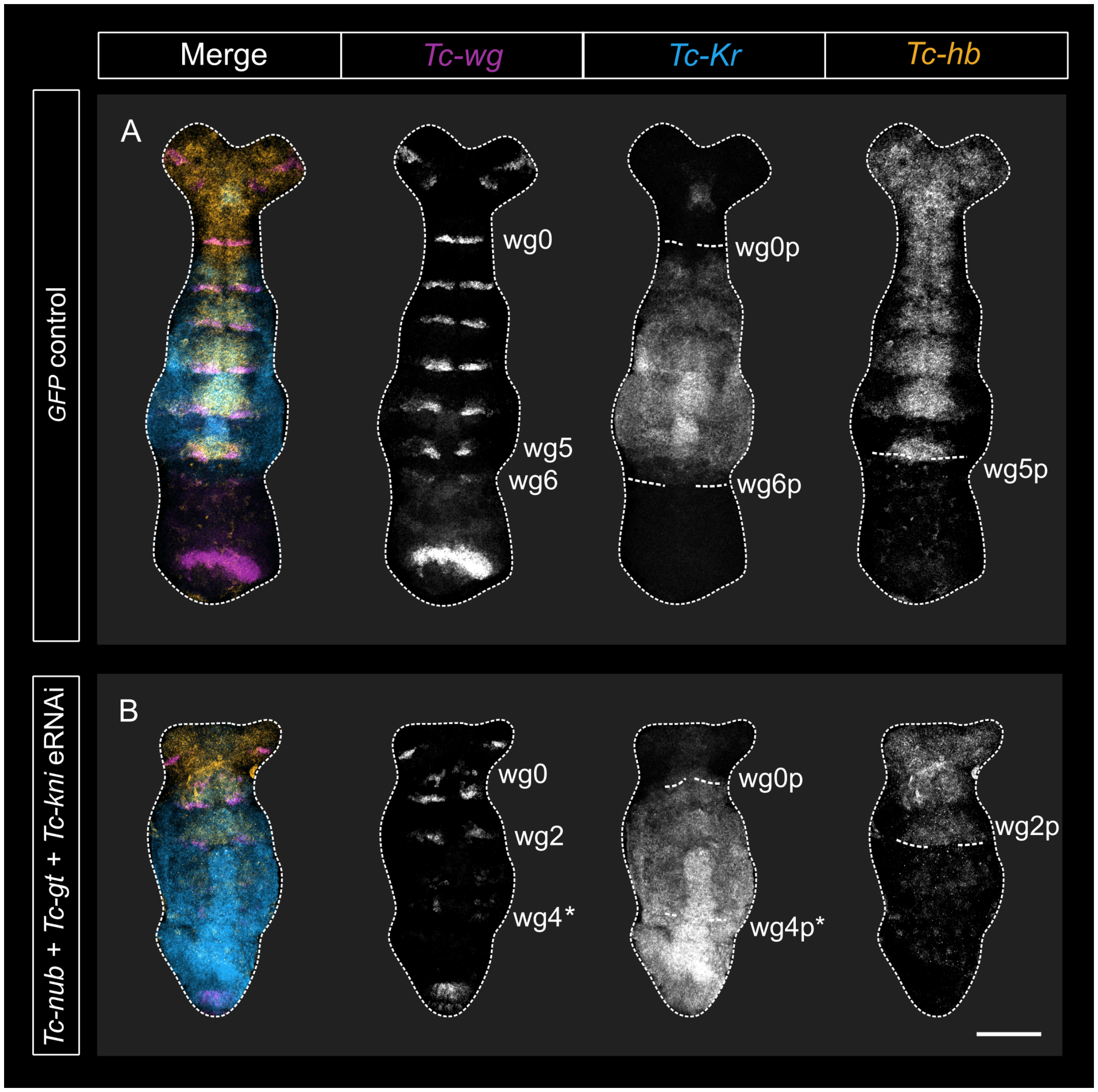

Fig. S9. Expression of *Tc-Kr,* but not *Tc-hb,* was expanded posteriorly after eRNAi against *Tc-nub* + *Tc-gt* + *Tc-kni*. (A) In *GFP* dsRNA-injected control embryos the expression of *Tc-hb* and *Tc-Kr* matched descriptions of wild-type expression (4/4) (Marques-Souza et al., 2008; Wolff et al., 1995). **(B)** In embryos injected with *Tc-nub* + *Tc-gt* + *Tc-kni* dsRNA, *Tc-Kr*, but not *Tc-hb,* expression was expanded compared to similarly staged wild type embryos (Marques-Souza et al., 2008; Wolff et al., 1995) (7/8 and 0/8 embryos showed expanded expression of *Tc-Kr* and *Tc-hb*, respectively). Embryos were fixed 16-17h AEL. All embryos were imaged using the same laser settings and brightness/contrast values were adjusted identically for all images. Anterior is to the top and ventral along the vertical midline of each embryo. wg0-6 = *Tc-wg* stripes 0-6; wg0-6p = posterior boundary of *Tc-wg* stripes 0-6. Asterisks are used to indicate *Tc-wg* stripes that are poorly patterned, where stripe designations are uncertain. Scale bar is 100 µM.

Development • Supplementary information

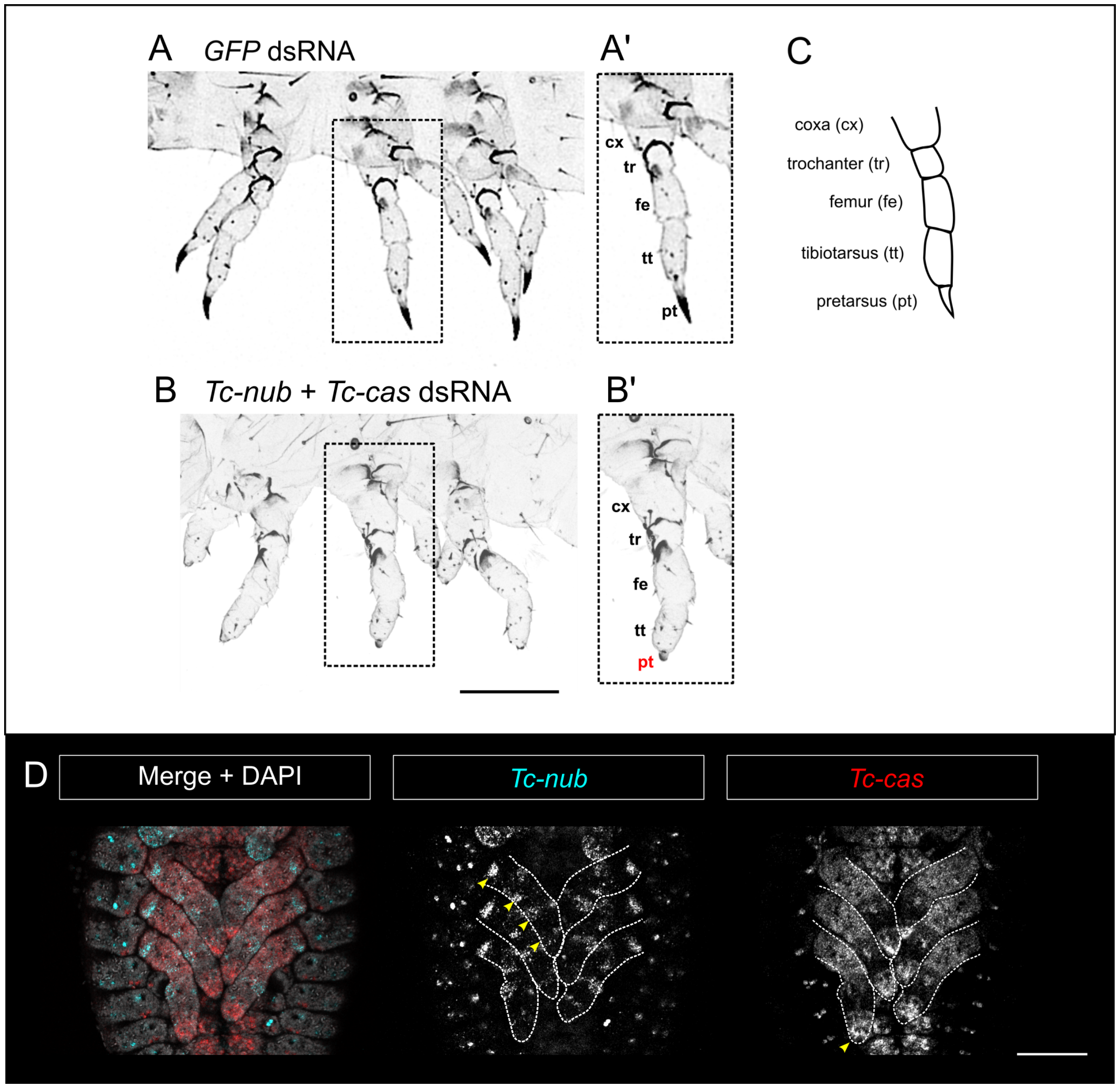

Fig. S10. Knockdown of *Tc-nub* + *Tc-cas* expression by eRNAi produced embryos with malformed pretarsi. (A) Embryos injected with *GFP* dsRNA (2 µg/µL) developed legs with normal external morphology (magnified and annotated in inset A'). **(B1-B2)** In embryos injected with *Tc-nub* + *Tc-cas* dsRNA (1 µg/µL each), the most distal leg segment - the pretarsus (pt) – failed to form normally (magnified and annotated in inset B'). **(C)** A graphical summary of the leg segments in *Tribolium*. **(D)** Expression of *Tc-nub* and *Tc-cas* in the developing legs. Yellow arrowheads mark rings of *Tc-nub* expression in the presumptive leg joints, and the expression of *Tc-cas* in the most distal portion of the leg, where the pretarsus will form. Images in A-B' and D are maximum projections of confocal z-stacks through cuticle preparations and dissected, flat mounted germbands, respectively. Scale bars are 100 µM.

Development • Supplementary information

Table S1. Cuticle phenotypes following pRNAi against *GFP*, *Tc-odd*, *Tc-nub* or *Tc-cas*. *Tc-odd* dsRNA was used as a positive control, and generated axial truncations similar to those reported previously (Choe et al., 2006). At 2 µg/µL, *Tc-nub* knockdown produced a range of cuticle phenotypes at low frequency, mostly affecting segment formation and patterning in the abdomen. Only the 'nub' phenotype was investigated in detail in this paper, as other phenotypes were not consistently identified in eRNAi experiments. The small percentage of cuticle defects observed after *Tc-cas* pRNAi at 2 µg/µL were not consistent between experiments and were not investigated further. 'N eggs' = number of eggs examined; 'N cuticles' = number of eggs that developed to the stage of cuticle formation. All percentages apply to the number of cuticles, not eggs. WT = wild type; 'nubs' = ectopic, ventrolateral cuticular protrusions on one or more abdominal segments (in these experiments, nubs were always limited to abdominal segment 1 (A1).

dsRNA injected	N eggs	N cuticles	% hatching	% WT	% nubs	% other defects
GFP (1 µg/µL)	404	**336**	94.3	100	0	0
Water	100	**83**	98.8	100	0	0
Tc-odd (1 µg/µL)	116	**49**	0	0	0	100
Tc-nub (1 µg/µL)	447	**198**	8.0	100	0	0
Tc-nub (2 µg/µL)	120	**68**	4.4	88.2	2.9	8.8
Tc-cas (1 µg/µL)	167	**68**	11.7	100	0	0
Tc-cas (2 µg/µL)	116	**45**	8.9	97.8	0	2.2

Table S2. Cuticle phenotypes following eRNAi against one or more of the genes *GFP*, *Tc-nub*, *Tc-cas*, *Tc-kni* and *Tc-gt*. Single knockdowns were carried out using 2 µg/µL of dsRNA, while all double and triple knockdowns used the component dsRNAs mixed to a final concentration of 1 µg/µL each. 'N eggs' = number of eggs injected and examined; 'N cuticles' = number of eggs that developed to the stage of cuticle formation. All percentages apply to the number of cuticles, not eggs. WT = wild type. 'nubs' and 'legs' both describe ectopic, ventrolateral cuticular protrusions on one or more abdominal segments, but the latter have joints and/or claws, while the former lack these features. The average (Avg) number of extra leg pairs refers to ectopic leg pairs forming on presumptive abdominal segments, and is calculated excluding embryos that lack any ectopic legs on the abdomen. Max = maximum.

Treatment (dsRNA injected)		N eggs	N cuticles	% Abdominal transformations			Avg / Max extra leg pairs
				'nubs'	legs	Total	
**Singles**	*GFP*	266	**171**	0	0	0	- / 0
	Tc-nub	148	**91**	12.1	0	12.1	- / 0
	Tc-cas	89	**43**	0	0	0	- / 0
	Tc-kni	45	**28**	0	0	0	- / 0
	Tc-gt	50	**36**	0	11.1	11.1	1 / 1
**Doubles**	*Tc-nub* + *Tc-cas*	38	**19**	15.8	0	15.8	- / 0
	Tc-nub + *Tc-kni*	93	**41**	43.9	24.4	68.3	1 / 1
	Tc-gt + *Tc-kni*	49	**28**	25	46.4	71.4	1 / 1
	Tc-nub + *Tc-gt*	95	**38**	31.6	50	81.6	1.3 / 2
**Triple**	*Tc-nub* + *Tc-gt* + *Tc-kni*	136	**35**	0	94.3	94.3	4.0 / 7

Development • Supplementary information

Table S3. Knockdown of *Tc-nub* and *Tc-kni* does not enhance the severity or penetrance of segment truncations observed after *Tc-gt* knockdown. Single knockdowns were carried out using 2 µg/µL of dsRNA, while double and triple knockdowns used component dsRNAs mixed to a final concentration of 1 µg/µL each. 'N cuticles' = number of cuticles examined. Embryos that are 'truncated' have at least one posterior segment deleted. The average (Avg) number of deleted segments is calculated across all cuticles examined (including those without axial truncations), and is rounded to the nearest whole number. Max = maximum.

Treatment (dsRNA injected)		N cuticles	% truncated	Avg / Max deleted segments
**Singles**	*GFP*	**42**	4.8	0 / 1
	Tc-gt	**34**	94.1	4 / 7
**Doubles**	*Tc-nub* + *Tc-kni*	**43**	7.0	0 / 1
	Tc-nub + *Tc-gt*	**22**	95.5	3 / 7
**Triple**	*Tc-nub* + *Tc-gt* + *Tc-kni*	**19**	94.7	3 / 7

Table S4. Sequences of clones used to generate dsRNA for *Tc-nub*, *Tc-cas*, *Tc-kni*, *Tc-gt* and *Tc-odd*. Sequences are presented as 5'-3', and correspond to the coding strand.

Gene	Sequence
Tc-nub	CGTCAGCACGGCAAAGAACGCAATCTGTGGCCATTTCAGTGCTCCAACCCTTAATGCCATCATCACCCCCGTGTAGTGACAGTGCCGACATATCACAGTGCAGTGCACG ATGCCCGAGTGAGCTCGCCCTCAATGAAGATATCGAATCTGGCGGATTTTAAAAGTGACACGGTCCGGAGGTGGGTCGAGTCGAACCCGTCACCCACCCTCCAGAACCT CGACACCCCCAAGTCCGCCAAGAACAACAACAACAACAACACCATCGAGTTCTGGCACCGGCAGACCGCCGACGACATGCCCATGTTGCTCCACCAGCCGCCCTCGCAC GTCCTCCTCGCCGGCCACGAGACCGACCAGCCCCTCGACTTCACCATGTCCAAGTTCAAGACCAAGGCGGCGACCACGGTGGCCTCCCAGCTCAAGAGCTTCGCCGCCC AGCAGCACATGATGCTCAACGGGGTGTACTTTAACCGCAACAACAACAAGGGTTTCACGCGCGGCTCGAGTCCGTCGAGTAGCTCCGAGGAGGAGG
Tc-cas	CCACATCAAAGACGAGCAACTCTCGCGCGACGGTTTCAAAAAATTCATGAAAAACGAAGTATGTCCCTTCGAAAAATGCCGTTTTTCCAAAGTTTGCAACCACATCCAT TGCATCCGCCCGCAGTGCAGCTACGTCTTGCACAGTTCAGGCCAACTGTTCTCGCACAAGCGAAAGCACGAACGCAAAGACAGCGAATTGGCTTATCGCAAGTACAAAC TGGCTCAAAGCATGATGAAGAGCCTCCAAGACGGCTCTCTAAACCCCTCATTTACACGCGACTACGAACAACAACTCGAAGGCTTAAACCTTTCGATGTTCAGCCAAAA CAGCAGCACTTCAAACGTCTCGGAAACGCTCAGCGAACGCAATTCGCCGGTTAGCTACGAAGAGAGCGAATCGGCGATTGATCTAACAGCGGGCGATTCAAATTTCGAA GAAACCAACTGGAACAGTGAGGATTTCTGGAAGAAGTACTGCCAGTTTGTGAGCCAACAAGACAGTTGCAGCGAAAAATGCGATTTTAACTATTCGGATCACTATCATT GCGTTGTGGAGAACTGTGAGATGGTTTTCAATGCGAAAGATGGGGTAAGGGAGCATGCTCGCAACCATGAACAGCAGGAAATCGTCACTGAGAATTACTTCACCACGGT GACGGGGCAGTGTGATGATGGGTGTATTTACCAGGATAAGGAGAAGCACTACCATTGCAATTGGGTGGGTATTGGAAAATTCAAACGCCAGAAATTAAATGCAAAATTG CAGGATAATTGTCGGGAGGTGATTTTGCCGAGCGATAAACCTTTCCGACGATTAGAACATTACAAAATGCATGAGTATTCGAGAAAATTGAGTTTGACGAAGGATCCAT TGACTATGACCCATTTGGCCACGTCCATCGATGGCATGTTTTGCAGGAAGAGGGGGAGACCGCCCAAAAATCGGGTCATTGAAGTGTGG
Tc-kni	ATGTGGCGAACCAGCAGCAGGATTTCATTTTGGAGCTTTCACGTGCGAAGGATGTAAGTCGTTCTTCGGGAGGTCGTACAACAACCTCAGCTCCATCTCGGAATGCAAA AACAATGGCGAGTGTGTCATCAATAAGAAGAATCGGACGGCTTGCAAAGCCTGCCGACTGCGGAAATGTCTGATGGTCGGTATGTCAAAAAGTGGCTCAAGATACGGCC GAAGATCAAACTGGTTCAAAATCCACTGTTTACTGCAAGAACAGCAACAGCAGCAACAAGCCGGTGGTGGTGGCATCCAAACCGGCCTCAAACCCCCCCAGAAAACCCC ATCCCCGCACCAACACCTCAGCCTCGGCCTCCTGAACCAGCCCTTCGGCCACCCCCTCATGCACCACATCACCAAAACCAAAGAAGAGCTCATGCTTCTTGGACTCGAC GAATACAAAAACTCCGCTTCGCCTTCGGTCAGTTCTCCCGAATCGCACAACTCCGACTCTTCGGTCGAGATCAGCGACGCTAGGAGAATACCCCTCTTCCCGGGACTCC TCCCGCCGACGTTTCTACCTCCTCATGGCCTGCTCTTCCCCCCCGGGTACCCGCCCCTGTACCCCGGCCTCCTCCAGCCCACCAACAACAACAACCGGCTGATGCGCAA CCACAACCCTGGAGTTGAAGCTTTCAACAAGCGGGTGTTCCTGGACGCGGTGTTGCAATCGCAGAGGTCTCCGACCCCGGAGGTGGAGGCGCCGCCGCCGGTGGCGTCG CCCCTGCAGGAGGACCCCATAGACTTGAGTATGAAGACTATGAGTGAGCGGGGGTCTTCACCGGCTCACAGTGATCGCTCGGATTCGGTTGTAACCGAGCAAGGTGCAG GCAGTGAAGCTGATGAGGAGAGCGATTGCGAGTCGGAGAAGGAGTTGAAGAGGATAAAGTTAGTCAGACCCGCGCCGTTGGACTTGACCACAAAAGTGTGA
Tc-gt	AATACAGCCCCGTCTCTAATAGCGATTCGGAGAACTCGGAAGTCTCCAGCAACTCCTACACGCCGAAAATAAAGAGCTGCCGCCCCTTCAAAGCCTACATCAAGGACCC CCTCACCCTGGCTCAGGGCTTAGTGAGCACGGAAATGCTATTGAAAAAGGATTCCTCCGAGGCGTTCAACGAATTCCGAACGAAAATCTTGGCACAAGTGCATGGGACC AACAACGGAACTAATAAAAACATGCGCAGGTTATCTACGACGACACAAAATAAAAACGATGATCCTAGTTATTGGGAGAAACGGCGGAAAAATAACGAAGCGGCGAAAA GATCCAGAGATGCCAGAAGAGCTAAAGAGGATGAGATTGCCATCAGGTGCGCCTTTCTCGAACGGGAAAACTGTCATCTGAAATTCGTCACGGATACGCTCAAGAAGGA GCTGGAGAAGCTACAG

Development • Supplementary information

Tc-odd	TGTCCGGCAAGTCAAACAGGAGGCTGACAGTACTTCGCCCCTTTTGACGCCCCCACACAACCCCGCCTATTCGCCGCCCCCGAGCTACTGCGATCCGTGGTACAACCCT<br>TATTACTACCAGTATTTGCAAAATGCGGCCCTGTATCACAAGTTGCAATTCAGGGGGCCCCCAAGGACCCCCCTTCAGCCTAAATTAGTGGCTGGGAAGCTCAAACCCA<br>AGCGACAATTCATCTGCAAGTATTGTAATCGACAATTCACTAAAAGCTACAACTTGTTGATCCATGAGAGGACACATACGGACGAAAGGCCCTACTCGTGTGACATCTG<br>TGGGAAGGCGTTCCGGCGTCAAGACCATCTGAGGGACCACAGGTAAGAAAAGAATATTTTAGCTTCTATTTTTTAATAGATTTTTTTAAAAGTACAGTTTAAATAACTT<br>TTTATTTTAAGTACTATTTAAATGTATTTTCAAGTAAAAATAGGTGGATTAATATTGTAAGAGGAAGTTTAAAACCAACCACAGTTTATTTCATCCCCTTGCTGCTAGC<br>TACCCCTATTTTAAATTTTTATAGTTGTACCTCTTACTTTTTTAAATGTTTTTGGAAAGTTGTTGAAAATCTTTAGCTAACTCCAAAATTAAAAAAGTGTCCACTTGGT<br>TTTGAGTTGGGGTTGATTTACTTTTGAAATATAAATTTCCGTAATTTTCGATTTTAATTTAATTAAACAAAAGAAAGTTTGTTTAGACAAAAAAGTACTATCGTCTATT<br>ATCGTCTTTAGTTTTCGAGTTAAAAACAAGAGTTAGTAAAATATTAAAAAATCATGTTTTTTATTATTGAAACAAAGACATTGTTAGGGATTTTTTTACGAATAATTTA<br>ATAGTGTTGTCAACGATTTATTTTAACCATTCGTTTAGCAGAAATAACATGAAGTTCTATTTTTTATTAATAGGAATTAAAGTTGGCTGACATTTTTGAAAAGCTTATT<br>TTTTTCTGATGTGAAAAATATTACATTTTTAAGTTAAAAACTCACTTTTTTTAAATATGTTTAAATAACTAAAAAATACGTTATTTTTTCCTGTTTTCAAGTGTTTTAG<br>TATGATTTTTAAACAATAACTGACTTACTTGGCATAATTTTGCCAAATTTTGCTAAAAACTGGCCAAATATTTTATTATTTTAACATTTTTTGTTACTTTGCAATATTT<br>AATAAAAAGTTACTAAATTACATAAAAATCGATATTTTTTTTCTTTTTTCTTGTTGGAGTGATTTAGCTCGTGTTATACGTTTTTTCACAACCTTTCGTTAAAATTGAA<br>CGAAAAATTACTTAATTTATTTTAAAATTATCTTACTTAAGTGCCACAGCACACATATTTGCAAAAATTTTACTTGACATGTAGAAAAATGTCTCGAAAATAACGAAAA<br>ATAATTTCTATTTTTGTAGATTTATCTGGTTTTACATTTGAAAAACTTTTTTGGAAATTTGGTGTAAAAAAGTTTGTTTTATAAGTGAAAAACATTTTTTTAATAAATG<br>GTTTTAAAATTCTTATCTAAGGAACGGTAAATACAAGATTAAAAAAAAAAAAAATTTCGCATATTTCGCTCAAATAAGTAAGCCAAAAGATTTCCATTTTGCAAAATTTT<br>GCTGAAAAGAGATGAAAAATTACTTAATTACTTGAATTATTTTGAGTTCAAACTCACTTAGGTGCTTTTAGAAATTTCTCTCTAAACGAAACAATAAATTTTCAAAAGT<br>TACAATAAATTTTATTACAGTTATTTTAAGCTGAAGAACATTTTTTTAAGTTAAAAACTCACTTTTTTCCGCAAAATTTTGTTTAAATAAATAACTAAATAAACTTTTT<br>CCTTTTTTGTTCTCAACCAAAAACACACTTATTTCGCATAATTTTGCTACAACATTTTTAACGTTTCTCGTTCTTTTTAGTGGTAAAACTGATAAAATCATCACTTTAC<br>AACAGTTAATTAAAAATTACCAGATTACATAAAAATTTAAGTTTAAGACGTGTTATACGCTAAATTTTTTTCGCATATTTTCGTTAAAATCTAACGATAAATTATTTAA<br>TTAAGTAATTAAAAATTACCTCATCTAAGAGCCATAATCTCACATATTTGCCAAAATGTATTATAAAAATTTTCCGAAAATAACGAACTTTCTCTTTGAATTTGGCTGT<br>TTTTTGCAAAATTGGAGTAAAAATCCAACCAATCAAGTAATTGTTTCATTAGATTTAGCTTGTTTTCTAAGTAAAAAGCAATTTTTTGTCAAATGGCCCTAAAACTTTT<br>ATCCAAGAGACGGTAAAGACAACATCATTATTTTTGTCTTTTTCAATAGGCTGAGCACATTTTTAATTTAAAAATTTCGCAAAATTTTGAAAATTTCTCTCAAATAAGT<br>AAGCCGAAAGATTCCTGAACACTATGAAAGATTACTTATTATTTCGTCCAGAACTCACTTACTTTGAGAAATTTCTAATAAAACAATAAATTTTCAATAAATCTCGAAA<br>GTTAGTTTTTTAATTATATTTTATATTTGAAAATTGCTATACATGCAATTAGTTAAAAATCATTTCGTATTAATTTTTTTCAATAGCCTTAGCACATTTTTAAGATAAA<br>AACTCACTTTCCTAAATTTTGTTTAAATAAATAACCAAAAAATACTTTCTTCGAGTATTTTAGAACGATCCTCAACCGAAAACTCTTTTATTTCGCATAATTTTGCTAC<br>CACAGGCCAAAAATGGCATTCTTTGTTGGGAATAATATTTCCGCTTGCTTTTTAGTGATAAAAATCATCACTTTGCAACATTTTATCACGAGTTACCTAATTACATAGA<br>AAATTACTGTTTTTTTAGCTTGGTTAGGGTGAAATTATTCTCGTAAACTTAATTAAGAGCTAAAAACTCGCTTATTTTTCGATTTAATTTTAAACTGTTGTTATTTTAT<br>GAATTCCAGGTACATCCACAGCAAGGAGAAGCCGTTCAAGTGCACGGAGTGCGGCAAGGGCTTCTGCCAGAGCCGGACTTTGGCGGTGCACAAAATCCTCCACATGGAG<br>GAATCCCCGCACAAGTGCCCCGTGTGCTCGCGCTCGTTCAACCAGCGCTCCAATCTCAAGACGCACTTGTTGACCCACACCGAGCGCCCCCTTGAGTGCACCCTCTGTG<br>CGCAGTTTTTCGCCAGCTACTCCGATCTCAAGACGCACGAGGCGCGCCATTGCCCCAAGGTGGAGGAAGTCGTCCAGCCCACGACGTGCCTCGATTTGACCAAGAAAAG<br>TGCAAGTCCCAAAGTCAAGCTGGGGTTCAGTATCGAGGACATTATGAAGCG

Development • Supplementary information
