# Supplementary material for: The neuroblast timer gene nubbin exhibits functional redundancy with gap genes to regulate segment identity in Tribolium
Source: Development. 2021 Aug 19;148(16):dev199719. doi: 10.1242/dev.199719 (PMC8406537; doi:10.1242/dev.199719)
Supplement: Reviewer comments [file develop-148-199719_review_history.pdf]

The neuroblast timer gene *nubbin* exhibits functional redundancy with gap genes to regulate segment identity in *Tribolium*

Olivia R. A. Tidswell, Matthew A. Benton and Michael Akam
DOI: 10.1242/dev.199719

Editor: Cassandra Extavour

Review timeline

Original submission:	20 April 2021
Editorial decision:	17 May 2021
First revision received:	24 June 2021
Accepted:	23 July 2021

Original submission

First decision letter

MS ID#: DEVELOP/2021/199719

MS TITLE: The neuroblast timer gene *nubbin* exhibits functional redundancy with gap genes to regulate segment identity in *Tribolium*

AUTHORS: Olivia R A Tidswell, Matthew A Benton, and Michael E Akam

I have now received all the referees reports on the above manuscript, and have reached a decision. The referees' comments are appended below, or you can access them online: please go to BenchPress and click on the 'Manuscripts with Decisions' queue in the Author Area.

The overall evaluation is positive and we would like to publish a revised manuscript in Development, provided that the referees' comments can be satisfactorily addressed. Please attend to all of the reviewers' comments in your revised manuscript and detail them in your point-by-point response. I especially encourage you to address the important points of consistently reporting sample sizes, proportion of animals with phenotypes of interest, statistical significance of phenotypes compared to negative controls, and efficacy and specificity of knockdown of target genes. If you do not agree with any of their criticisms or suggestions explain clearly why this is so.

We are aware that you may currently be unable to access the lab to undertake experimental revisions. If it would be helpful, we encourage you to contact us to discuss your revision in greater detail. Please send us a point-by-point response indicating where you are able to address concerns raised (either experimentally or by changes to the text) and where you will not be able to do so within the normal timeframe of a revision. We will then provide further guidance. Please also note that we are happy to extend revision timeframes as necessary.

Reviewer 1

Advance summary and potential significance to field

There are several areas where the authors contribute important novel functional insights: Most importantly, they provide evidence that the temporal control acting in neuroblasts was at least partially taken over for spatial patterning of the embryo. Next, they provide the first (to my

© 2021. Published by The Company of Biologists under the terms of the Creative Commons Attribution License (https://creativecommons.org/licenses/by/4.0/).

knowledge) model explaining the genetic control of the distinction of the insect body into three tagma (head, thorax and abdomen). Finally, they add a large body of valuable data to our knowledge on the segmentation gene network in Tribolium (including the unexpected and novel synergy between gt, kni and nub).

Comments for the author

The Drosophila paradigm of insect embryonic patterning is based on spatial patterning within a given field of cells. However, most insects and other animals pattern their trunk during a posterior elongation and patterning process, which requires temporal control to realize according spatial gene expression patterns. It has remained an open question, in how far spatial and temporal patterning processes are similar or divergent and in how far they are based on the same gene sets and/or interactions. A paradigmic example for temporal pattern is provided by the temporal expression of transcription factors in Drosophila neural stem cells (neuroblasts). Interestingly, the temporal pattern of expression of Hb, Kr, nub and cas in neuroblasts is reflected in their anterior to posterior patterns of expression in the Drosophila embryo and Hb and Kr have important segmentation functions. This finding fueled speculations that the more ancestral neural temporal cascade would have been adopted for segmentation. However, several attempts to detect a function in segmentation for nub and cas have failed.

Tidswell et al. now provide very convincing evidence of an important role of nub in the embryonic patterning gene network in the red flour beetle Tribolium castaneum. Specifically, they find a crucial role in the regulation of segment identities via Hox gene regulation. First, they very carefully and comprehensively describe the expression patterns of nub and cas relative to other relevant genes. Then they use RNAi to show that neither nub nor cas show a strong phenotype on their own. However, in double and triple RNAi experiments they convincingly show a synergy among nub with giant and knirps to to regulate proper abdominal Hox gene expression. The authors assign the observed effects to the joint repression of Kr, which in turn represses posterior Hox genes. The authors discuss their data in terms of the segmentation gene network in Tribolium and models of the evolutionary co-option of the neuroblast timer network to trunk segmentation.
This is an excellent study, which is based on very convincing, extensive and well controlled data. There are several areas where the authors contribute important novel functional insights: Most importantly, they provide evidence that the temporal control acting in neuroblasts was at least partially taken over for spatial patterning of the embryo. Next, they provide the first (to my knowledge) model explaining the genetic control of the distinction of the insect body into three tagma (head, thorax and abdomen). Finally, they add a large body of valuable data to our knowledge on the segmentation gene network in Tribolium (including the unexpected and novel synergy between gt, kni and nub). The paper is rich in data (side aspects are carefully and comprehensively analyzed, as well), which are displayed in excellent figures and findings and interpretation are visualized in very nice schematics. The piece is very well written.

Typos and stuff:
Fig legend 1: A and P do not need to be defined – these words are given in full in the figure.
Line 339: I guess you wanted to refer to panel I – not G Fig legend 5/6: delete “overleaf”
Line 520: “gene:tagma” or “gene-tagma”?
Fig. legend has different reference style [number] rather than author/date

<u>Reviewer 2</u>

Advance summary and potential significance to field

The manuscript by Tidswell et al., examines the roles of nubbin (nub) and castor (cas) in axial patterning in Tribolium. Hb, Kr, Nub and Cas act sequentially in a timing mechanism that regulates neuroblast differentiation in Drosophila and some vertebrate neurons. It has been hypothesized that this neuroblast timer pathway was co-opted for axial patterning in arthropods. The authors provide nice in situ hybridization data showing the sequential activation of Hb, Kr, nub and cas in the posterior growth zone (the SAZ) of the Tribolium embryo. Knockdown experiments support a role for nub in regulating segment identity semi-redundantly with the gap genes knirps and giant. These genes regulate hox expression by repressing Kr which, in turn, regulates hox expression. The

© 2021. Published by The Company of Biologists under the terms of the Creative Commons Attribution License (https://creativecommons.org/licenses/by/4.0/).

repression of Kr by Nub is a feature of the neuroblast timer pathway. Thus, these functional data, along with the gene expression data, support the hypothesis that the neuroblast timer pathway has been co-opted for axial patterning in arthropods.
Overall, the data are of high quality and the manuscript is clearly written. However, there are a number of issues that need to be addressed before the manuscript would be ready for publication.

Comments for the author

Many of the knockdown phenotypes are observed at low frequency. However, there are no statistical tests to determine if these frequencies are different from negative controls. In fact, there do not appear to be tests for statistical significance in any of the experiments. The authors should provide an appropriate statistical test for all of the experiments and report the results in the Figure legends.
In Figure 5, how many triple knockdown embryos were analyzed and what percent exhibited loss of Ubx and adb-A expression? The authors are inconsistent in indicating how many embryos were examined in each experiment, how many independent experiments were performed and what percentage of embryos exhibited a given phenotype. These numbers should be provided for all experiments.
In figure 8, the authors should more clearly denote which regulatory interactions have been empirically demonstrated versus hypothesized or inferred (e.g. hb repressing Nub and Cas in the Tribolium SAZ). In fact the repression of Nub and Cas by Hb are major features of the author's model and demonstrating this by knockdown of Hb would greatly strengthen the author's conclusions.

Reviewer 3

Advance summary and potential significance to field

Very interesting manuscript! The authors document an unexpected role at least a part of the Drosophila neuroblast timer in the SAZ of Tribolium and reveal a number of other novel regulatory interactions in the progressive specification of segment identity in the posterior. The authors carefully (and beautifully) describe the sequential appearance of nubbin and cas relative to known gap gene expression patterns in the posterior. They find that the function of nubbin was previously obscured by redundant function with gap genes with overlapping expression domains in the posterior. Knockdown of gt, kn and nub result in a loss of function of Ubx and abdominal A and appearance of additional thoracic appendages – limited by the failure of gt knockdowns to form all segments. This triple knockdown reveals a role for these genes in repressing Kr , and for Kr as a repressor of posterior Hox genes.

The manuscript adds important new discoveries to the regulatory control of patterning in a sequential segmentation, raises as many questions as it answers, and should be of interest to a broad segment of developmental biologists.

Comments for the author

I recommend publication after attention to the minor details below:
My major problem with the manuscript as currently written is the role of cas. The authors have found that cas expression initiates in the SAZ after that of hb/Kr/nubbin as expected from the neuroblast timer, but they have not shown a function for it in patterning the posterior, and its expression pattern itself is problematic for a timer model. Also, as far as I can see, the expected activation of nubbin by Kr, and cas by nubbin have not been documented? Is the nub/cas timing co-option or coincidence?
Thus, it would seem prudent that they tune down their proclamation of a conserved neuroblast timer just a bit and include a more realistic discussion – perhaps that the timer was recruited and cas function subsequently lost?
Also somewhat left behind in the discussion in the sequential appearance of a second hb domain- this expression is included in the model (circles) in Fig. 2 but never addressed again, even as a discussion point. This too argues for a variant of the Drosophila neuroblast timer..

© 2021. Published by The Company of Biologists under the terms of the Creative Commons Attribution License (https://creativecommons.org/licenses/by/4.0/).

Fig. 3. It is confusing that the figure columns are labeled by the number of segmental wg stripes, but the embryos themselves are labeled with a different number. It would certainly be much simpler to follow the text and figure if the wg stripes were simply labeled consecutively and not ask the reader -particularly those unfamiliar with how to translate parasegments into segments (like me)- to have to translate.
The text says posterior boundary of nubbin defined by wg 12 but figure says wg 12p
The legend suggests the diagrams in panel l are confocal sections?
A brief clarification as to why the reader should ignore all expression anterior to PS4 would be helpful. The expression in H1 and older expands throughout the segment, beyond the offered neuroectoderm explanation. Cas also seems absent from neuroectoderm and abundant in legs despite the statement that its expressed in neuroectoderm?
“Together, these data suggest that Tc-nub, Tc-Kr and Tc-kni redundantly repress Tc-Kr expression and that in their absence, Tc-Kr expression expands into the abdominal primordia. “. I believe Tc-Kr is an error in the first clause of this sentence, and it should read Tc-gt?

Methods:
RNA interference: what measures did the authors take to insure their targeted knockdowns were not interfering with other genes (particularly the nubbin oogenesis phenotype, but potentially other issues). Frequently authors are expected to perform RNAi with two different constructs, or to minimally report that their fragments at least computationally are not predicted to affect off target sites.
Did the authors really inject halocarbon oil into the eggs or is part of this sentence missing?
Were these embryos subsequently removed from their membranes with forcing through a needle as above, or did the ice cold MEOH remove as in flies?

First revision

Author response to reviewers' comments

Reviewer 1

We thank reviewer one for the positive feedback, and also for identifying the following minor typos, which have been corrected:

Fig legend 1: A and P do not need to be defined - these words are given in full in the figure.
This annotation was meant to describe Figure 1 A1, and has been shifted to this part of the legend.

Line 339: I guess you wanted to refer to panel I - not G.
Corrected.

Fig legend 5/6: delete “overleaf”
Deleted.

Line 520: “gene:tagma” or “gene-tagma”?
Altered to gene-tagma.

Fig. legend has different reference style [number] rather than author/date.
Format has been changed to author/date reference style.

Reviewer 2

1. Many of the knockdown phenotypes are observed at low frequency. However, there are no statistical tests to determine if these frequencies are different from negative controls. In fact, there do not appear to be tests for statistical significance in any of the experiments.

© 2021. Published by The Company of Biologists under the terms of the Creative Commons Attribution License (https://creativecommons.org/licenses/by/4.0/).

The authors should provide an appropriate statistical test for all of the experiments and report the results in the Figure legends.
We agree that statistical analysis could help to bolster many of the findings in the paper. We have performed Fisher's exact tests to determine the statistical significance of differences in the proportions of cuticles developing nubs, forming cuticle or hatching in *nub, cas and GFP RNAi treatments.* The results of these tests have been included in the text as indicated below:

"Specifically, 2.9% (pRNAi, N=68) and 12.1% (eRNAi, N=91) of cuticles displayed a 'nub' (an ectopic protrusion of cuticle, lacking joints or claws) on either side of segment A1; a phenotype that was never observed in GFP pRNAi (N=336, Fisher's exact test: p=0.02798) or eRNAi (N=171, Fisher's exact test: $p=5.853x10^{-6}$) controls (Fig 4, B; Tables S1 and S2)."

"Only ~40-60% of eggs developed cuticle after 1 µg/µL Tc-nub or Tc-cas pRNAi, compared to 83% of eggs in GFP controls (Fisher's exact test: $p<2.2 x10^{-6}$; Fig S4, Tables S1 and S2)."

"Specifically, 8-12% of embryos that develop cuticle after 1 µg/µL Tc-nub or Tc-cas pRNAi go on to hatch, compared to 94% in GFP controls (Fisher's exact test: $p<2.2 x10^{-6}$; Fig S4, Table S1)."

For determining the statistical significance of differences in frequencies of abdominal transformations or axial truncations between eRNAi treatment groups, we have performed Bayesian logistic regressions. The results of statistical analyses on these regressions are reported in the graphs and legends of Fig 4 and the new Fig S7 (text copied below).

Figure 4: *"A Bayesian logistic regression of abdominal transformation frequency on eRNAi treatment indicated that eRNAi treatments differed significantly in their odds of generating abdominal transformations (χ^2 (df=7) = 314.7, $p<2.2x10^{-16}$). A Tukey post-hoc test was used to determine significant differences between groups, indicated as the letters on top of each column; treatments marked with different letters are significantly different from each other at the p<0.02 level."*

Figure S7: *"A Bayesian logistic regression of axial truncation frequency on eRNAi treatment indicated that eRNAi treatments differed significantly in their odds of generating axial truncations (χ^2 (df=4) = 151.84, $p<2.2x10^{-6}$). A Tukey post-hoc test was used to determine significant differences between groups, indicated as the letters on top of each column; treatments marked with an 'a' are significantly different from those marked 'b' at the $p<1x10^{-5}$ level."*

We have also added a short section on statistical analyses to the end of the methods.

2. In Figure 5, how many triple knockdown embryos were analyzed and what percent exhibited loss of* Ubx *and* adb-A *expression? The authors are inconsistent in indicating how many embryos were examined in each experiment, how many independent experiments were performed and what percentage of embryos exhibited a given phenotype. These numbers should be provided for all experiments.

We agree that this was an oversight, and have included the number of embryos examined, and the proportion showing the relevant phenotype, in the legends of Figure 5 and Figure 6. Note that collecting and staining embryos after eRNAi is challenging, as they must be manually dissected from the eggshell. Combined with the high early mortality of triple knockdown embryos, this necessarily results in a very small pool of embryos to examine for ISH after eRNAi.

3. In Figure 8, the authors should more clearly denote which regulatory interactions have been empirically demonstrated versus hypothesized or inferred (e.g.* Hb *repressing* nub *and* cas *in the Tribolium SAZ). In fact, the repression of* nub *and* cas *by* Hb *are major features of the author's model and demonstrating this by knockdown of* hb *would greatly strengthen the author's conclusions.

We agree that this distinction was not clear enough in the previous version of the figure. The

© 2021. Published by The Company of Biologists under the terms of the Creative Commons Attribution License (https://creativecommons.org/licenses/by/4.0/).

interaction between Hb and *nub/cas* in Figure 8B2 is now represented by a dotted line, to indicate that it has been inferred rather than empirically demonstrated, as described in the legend. Other previously-dotted lines (namely the "activating interactions" in B1) have been changed back to solid lines, to make the distinction more obvious.

We have also altered the colour scheme of the arrows in Figure 8 B1 and B2 to be more colour blind friendly.

We agree that the addition of *hb* knockdown data to the paper would be very informative, and were in fact planning on performing this knockdown before COVID prevented access to the lab. Unfortunately, the lead author of the paper has now moved on to a new job in another country, and we are not in a position to complete these experiments.

Reviewer 3

1. My major problem with the manuscript as currently written is the role of* cas*. The authors have found that* cas *expression initiates in the SAZ after that of* hb/Kr/nubbin *as expected from the neuroblast timer, but they have not shown a function for it in patterning the posterior, and its expression pattern itself is problematic for a timer model. Also, as far as I can see, the expected activation of* nubbin *by* Kr*, and* cas *by* nubbin *have not been documented? Is the* nub/cas *timing co-option or coincidence? Thus, it would seem prudent that they tune down their proclamation of a conserved neuroblast timer just a bit and include a more realistic discussion - perhaps that the timer was recruited and* cas *function subsequently lost? Also somewhat left behind in the discussion in the sequential appearance of a second* hb *domain- this expression is included in the model (circles) in Fig. 2 but never addressed again, even as a discussion point. This too argues for a variant of the* Drosophila *neuroblast timer.

We agree that the discussion as originally written glossed over the additional *hb* domain and the lack of a clear function for *cas*, and have altered the text in two places to highlight them (details below):

a) In the results section "The neuroblast timer genes are expressed sequentially in the SAZ" we have added the following sentence to draw attention to the secondary domain of *hb*:
"This re-expression of hb after cas is not observed during neurogenesis in either Drosophila or Tribolium (Biffar and Stollewerk, 2014; Doe, 2017), so is a distinctive feature of the SAZ."

b) In the discussion section "Co-option of the neuroblast timer series for axial patterning in insects" we have added a paragraph highlighting discrepancies in the network structure and function between the SAZ and neuroblast series.

"Beyond the broad similarities presented in this paper, we have also identified some key differences in the order of expression and function of neuroblast timer genes in neuroblasts and in the SAZ. Firstly, hb is expressed in the SAZ after cas, something that is not observed in neuroblasts. This posterior domain of hb is conserved in a range of insect lineages (Jaeger, 2011; Liu and Kaufman, 2004a; Marques-Souza et al., 2008; Mito et al., 2005), and has been hypothesised to influence the duration of segmentation (Nakao, 2016). It seems likely, then, that it is a significant component of the timer network in the SAZ. Furthermore, although we found a clear role for nub in regulating axial identities, we have found no such role for cas. Unlike the other neuroblast timer genes, expression of cas in the SAZ of Tribolium is modulated in a complex pair-rule pattern, arguing against its regulating axial identity across a broad, continuous region of the SAZ. The function of the 'gap-like' domain of cas expression in Drosophila also remains mysterious, as Dm-cas mutants appear normal outside of their neural defects (Mellerick et al., 1992). It may be that cas, like nub, acts redundantly with other genes to exert its influence on axial identity; that it has lost the ability to regulate axial identity in Tribolium and Drosophila; or that it never had such a role. Analysis of cas expression and function in the SAZs of other insect species may help to distinguish between these possibilities."

Although it is true that we have not documented activation of *nub* by Kr, and of *cas* by nub, we don't see this as an issue for co-option theory. Firstly, our proposal does not hinge on the

© 2021. Published by The Company of Biologists under the terms of the Creative Commons Attribution License (https://creativecommons.org/licenses/by/4.0/).

networks being identical – we would not be surprised to see extensive modification in the interactions between genes. Secondly, there is some disagreement as to whether these 'activating interactions' exist, and if they do, whether they are important, in neuroblasts. To highlight this, we have altered the colour of the "activating" arrows in Figure 8 B2 to grey, and added the following statement to the legend:
"Note that, at least in the neuroblast timer network, repression between network components seems to be more significant for network dynamics than activation (Averbukh et al., 2018)."

2. [In] Fig. 3. it is confusing that the figure columns are labeled by the number of segmental wg stripes, but the embryos themselves are labeled with a different number. It would certainly be much simpler to follow the text and figure if the **wg** ***stripes were simply labeled consecutively and not ask the reader - particularly those unfamiliar with how to translate parasegments into segments (like me)- to have to translate.***

This is a valid point (I myself nearly always have to use diagrams to translate parasegments to segments). However, we would prefer not to alter our labelling system for individual *wingless* stripes. We believe that it is important that the label for each *wingless* stripe links it explicitly to a parasegment, such that the first *wingless* stripe to form in the embryo (at the posterior of the PS0) is labelled as wg0. *wingless* is expressed at the posterior boundary of each parasegment, and the vast majority of segmentation genes are also expressed in parasegmental patterns. This designation therefore has the most biological relevance. By comparison, referring to the first trunk *wingless* stripe as wg1 provides no or misleading information about its relationship to the parasegmental/segmental pattern.

We understand that the previous combination of our stripe labelling system and embryo staging system may confuse readers. To simplify this, we have altered the labelling of the columns in Figure 3 so that stages are named according to the identity of their most recently-formed *wingless* stripe. This means that the stage labelling is more clearly linked to the patterning of parasegments. We have also added some additional explanation of our staging/spatial markers system:

In the first paragraph of the results section "Expression of *Tc-nub* and *Tc-cas* in relation to segment patterning", we have added:

"Each Tc-wg *stripe marks the posterior boundary of a parasegment (PS), and has been assigned a number that reflects its relationship to that parasegment (e.g., wg6 sits at the posterior of PS6; note that the first trunk* Tc-wg *stripe is designated wg0, as it sits at the posterior of PS0)."*

In the legend of Figure 3, we have added:
"Column headers indicate the identity of the most recently formed Tc-wg *stripe as a proxy for developmental stage"*

4. The text says posterior boundary of **nubbin** ***defined by*** **wg** ***12 but figure says*** **wg** ***12p.***

We realise that the description of the *Tc-nub* 'overlapping' with wg12 did not clearly communicate that its posterior boundary overlaps with the posterior of that *wingless* stripe – we have altered the text to say "the posterior boundary of *Tc-nub* eventually overlaps with the posterior boundary of wg12 (wg12p; Fig 3, F1-J1)". This makes the connection with what is shown in the figure more explicit.

5. The legend suggests the diagrams in panel l are confocal sections?

We have changed the start of the sentence ("All images") to "Excluding the diagrams in panels L1-L2, all images…".

6. A brief clarification as to why the reader should ignore all expression anterior to PS4 would be helpful. The expression in H1 and older expands throughout the segment, beyond the offered neuroectoderm explanation. **Cas** ***also seems absent from neuroectoderm and abundant in legs, despite the statement that it's expressed in neuroectoderm?***

© 2021. Published by The Company of Biologists under the terms of the Creative Commons Attribution License (https://creativecommons.org/licenses/by/4.0/).

In this section we are primarily concerned with which parasegments express *nub/cas* while they are initially being patterned, *i.e.* while their primordia are still in the SAZ. Hence the phrasing: "*Tc-nub* is therefore expressed in the SAZ during the patterning of PS4-PS12". It is true, however, that later expression of *Tc-nub* seems to extend beyond the neurectoderm.
To highlight this, we have altered the sentence at the end of the section on expresson to read:
"Tc-nub later becomes broadly expressed in the ectoderm outside of the SAZ, with slightly stronger expression in the developing neurectoderm (Biffar and Stollewerk, 2014)."

Thank you also for catching the mistake regarding expression of *nub* and *cas* in neurectoderm – you are correct that *nub*, but not *cas*, is expressed in the neurectoderm, while both genes are expressed in neuroblasts. To follow up on the previous altered sentence, we have added one that reads:

"Tc-nub and Tc-cas are expressed in neuroblasts (Biffar and Stollewerk, 2014) and the limb buds (Figure 3, G1-K1 and J2-K2)."

7. "Together, these data suggest that* Tc-nub, Tc-Kr *and* Tc-kni *redundantly repress* Tc-Kr *expression, and that in their absence,* Tc-Kr *expression expands into the abdominal primordia". I believe* Tc-Kr *is an error in the first clause of this sentence, and it should read* Tc-gt*?

This was an error, and has been corrected.

8. RNA interference: what measures did the authors take to ensure their targeted knockdowns were not interfering with other genes (particularly the nubbin oogenesis phenotype, but potentially other issues). Frequently authors are expected to perform RNAi with two different constructs, or to minimally report that their fragments at least computationally are not predicted to affect off target sites.

To bolster confidence in the specificity of our knockdown experiments, we have performed computational analyses of all dsRNA fragments using the online tool Deqor, and added the following sentence to the methods section titled 'RNA Interference':
"All dsRNA fragments used were computationally predicted to have a low potential for off-target gene silencing using the default search parameters of Deqor version 3.0 (i.e., the quality score of all potentially cross-silencing siRNAs was >5) (Henschel et al., 2004)."
We have also added the sequences for the dsRNA fragments used in Table S3.

We have clones for two, non-overlapping fragments for RNAi against *nub* (targeting either the 3' or 5'UTR) but have performed the vast majority of our experiments with only one – specifically, the fragment targeting the 5' UTR. An initial sample of dsRNA generated from both constructs was provided by our collaborator, Andrew Peel. One of his students has previously performed pRNAi knockdowns using dsRNA targeting the 3' UTR of *nub,* and found no phenotypic effects on the cuticle. This may be because this fragment has a weaker knockdown effect, or because the very subtle cuticle phenotype was missed. We began to redo these experiments at the end of my PhD, once it became clear that the 5' dsRNA influenced abdominal patterning, but were prevented from completing them by the onset of the pandemic. The fact that our *Tc-nub* dsRNA has little effect on its own, but a stronger effect in combination with *Tc-kni* and *Tc-gt*, does suggest to us that it is exerting its influence on abdominal patterning through specific interaction with the gap gene network – something that we consider to be an unlikely outcome of off-target effects. We concede that the specificity of the oogenesis phenotype is less clear, although is supported by our finding that *Tc-nub* is expressed in the ovary. Unfortunately, it will not be possible to complete knockdown experiments using additional *Tc-nub* and *Tc-cas* fragments, as the main author has now moved on to a new position in another country and the final author officially retired.

9. Did the authors really inject halocarbon oil into the eggs or is part of this sentence missing? Were these embryos subsequently removed from their membranes with forcing through a needle as above, or did the ice cold MEOH remove as in flies?

The sentence as written – *"Eggs were injected… **in** a 1:1 mix of Halocarbon oil 700 and Halocarbon oil 27"* is a little confusing, and we have amended it to read *"Eggs were covered with a 1:1 mix of Halocarbon oil 700 and Halocarbon oil 27….and dsRNA was injected into the anterior pole".*

© 2021. Published by The Company of Biologists under the terms of the Creative Commons Attribution License (https://creativecommons.org/licenses/by/4.0/).

Introducing a hole into the membrane during eRNAi makes it very difficult to remove the membranes using methanol shock/forcing them through a needle. For this reason, both the chorion and vitelline membrane was removed manually after fixation, as described in the paragraph below (from the same methods section):

"For fixation, injected embryos were aged for the appropriate length of time then injected with PBT + 10 % formaldehyde (v/v) and left to fix at room temperature for one hour. They were then transferred using an eyelash hair to Eppendorf tubes and fixed for an additional hour in a 1:1 mix of heptane and PBT + 4 % formaldehyde (v/v). The aqueous layer was removed, and 100 % ice-cold methanol added. Germbands were manually dissected away from the remainder of the yolk, chorion and vitelline membrane in PBS, and then stored in 100 % methanol at -20 °C until required. "

Second decision letter

MS ID#: DEVELOP/2021/199719

MS TITLE: The neuroblast timer gene *nubbin* exhibits functional redundancy with gap genes to regulate segment identity in *Tribolium*

AUTHORS: Olivia R A Tidswell, Matthew A Benton, and Michael Akam
ARTICLE TYPE: Research Article

I am happy to tell you that your manuscript has been accepted for publication in Development, pending our standard ethics checks.

Reviewer 1

Advance summary and potential significance to field

The authors have done a very good job to make the manuscript better - they have thoughtfully answered all reviewer´s requests.

Comments for the author

The authors have done a very good job to make the manuscript better - they have thoughtfully answered all reviewer´s requests.

© 2021. Published by The Company of Biologists under the terms of the Creative Commons Attribution License (https://creativecommons.org/licenses/by/4.0/).
